# Interactome Mapping Reveals the Evolutionary History of the Nuclear Pore Complex

**DOI:** 10.1371/journal.pbio.1002365

**Published:** 2016-02-18

**Authors:** Samson O. Obado, Marc Brillantes, Kunihiro Uryu, Wenzhu Zhang, Natalia E. Ketaren, Brian T. Chait, Mark C. Field, Michael P. Rout

**Affiliations:** 1 The Rockefeller University, New York, New York, United States of America; 2 School of Life Sciences, University of Dundee, Dundee, Scotland, United Kingdom; MIT, UNITED STATES

## Abstract

The nuclear pore complex (NPC) is responsible for nucleocytoplasmic transport and constitutes a hub for control of gene expression. The components of NPCs from several eukaryotic lineages have been determined, but only the yeast and vertebrate NPCs have been extensively characterized at the quaternary level. Significantly, recent evidence indicates that compositional similarity does not necessarily correspond to homologous architecture between NPCs from different taxa. To address this, we describe the interactome of the trypanosome NPC, a representative, highly divergent eukaryote. We identify numerous new NPC components and report an exhaustive interactome, allowing assignment of trypanosome nucleoporins to discrete NPC substructures. Remarkably, despite retaining similar protein composition, there are exceptional architectural dissimilarities between opisthokont (yeast and vertebrates) and excavate (trypanosomes) NPCs. Whilst elements of the inner core are conserved, numerous peripheral structures are highly divergent, perhaps reflecting requirements to interface with divergent nuclear and cytoplasmic functions. Moreover, the trypanosome NPC has almost complete nucleocytoplasmic symmetry, in contrast to the opisthokont NPC; this may reflect divergence in RNA export processes at the NPC cytoplasmic face, as we find evidence supporting Ran-dependent mRNA export in trypanosomes, similar to protein transport. We propose a model of stepwise acquisition of nucleocytoplasmic mechanistic complexity and demonstrate that detailed dissection of macromolecular complexes provides fuller understanding of evolutionary processes.

## Abstract 2

Dissection of the nuclear pore complex—an ancient eukaryotic molecular machine—exposes a fundamental divergence in structure and function between yeast and humans versus trypanosomes and provides insights into the evolution of the nucleus.

## Author Summary

Much of the core architecture of the eukaryotic cell was established over one billion years ago. Significantly, many cellular systems possess lineage-specific features, and architectural and compositional variation of complexes and pathways that are likely keyed to specific functional adaptations. The nuclear pore complex (NPC) contributes to many processes, including nucleocytoplasmic transport, interactions with the nuclear lamina, and mRNA processing. We exploited trypanosome parasites to investigate NPC evolution and conservation at the level of protein–protein interactions and composition. We unambiguously assigned NPC components to specific substructures and found that the NPC structural scaffold is generally conserved, albeit with lineage-specific elements. However, there is significant variation in pore membrane proteins and an absence of critical components involved in mRNA export in fungi and animals (opisthokonts). This is reflected by the completely symmetric localization of all trypanosome nucleoporins, with the exception of the nuclear basket. This architecture is highly distinct from opisthokonts. We also identify features that suggest a Ran-dependent system for mRNA export in trypanosomes, a system that may presage distinct mechanisms of protein and mRNA transport in animals and fungi. Our study highlights that shared composition of macromolecular assemblies does not necessarily equate to shared architecture. Identification of lineage-specific features within the trypanosome NPC significantly advances our understanding of mechanisms of nuclear transport, gene expression, and evolution of the nucleus.

## Introduction

In order to uncover the origins of eukaryotes, we must understand how their defining organelle, the nucleus, and its delineating nuclear envelope (NE) arose. The NE provides a barrier that defines the nucleoplasm and cytoplasm, and this discrimination represents a major evolutionary transition [1]. The sole mediators of macromolecular exchange between the nucleoplasm and cytoplasm are nuclear pore complexes (NPCs) [2]. Each NPC is a ~50 MDa, cylindrical, and octagonally symmetric structure comprised of nearly 500 proteins, these being multiple copies of ~30 different nucleoporins (Nups) [3–8]. There are three major Nup classes: pore membrane proteins (Poms), core scaffold Nups, and FG-repeat Nups. Poms contain *trans*-membrane domains (TM) that serve to anchor the NPC to the NE, whilst the core scaffold Nups are major structural components and also interact with the NE and Poms. The core scaffold consists of two inner rings sandwiched between two outer rings and is comprised of three groups of proteins containing only two major folds: α-solenoids and β-propellers, or an N-terminal β-propeller followed by an α-solenoid [9]. Interestingly, vesicle coat proteins, including clathrin/adaptin, COPI, and COPII, share architectural characteristics with the components of the outer ring Nups of the NPC, suggesting a common ancestry between the endomembrane trafficking system and the NPC; this is known as the protocoatomer hypothesis [9–12]. In addition to providing the structural core of the NPC, scaffold Nups provide a platform for anchoring FG-Nups, natively disordered proteins characterized by domains enriched in phenylalanine-glycine (FG) repeats and responsible for the selective permeability barrier to nucleocytoplasmic transport. In animals and fungi, a large subset of FG-Nups have a biased distribution across the NPC, with ~30% predominantly at either the nucleoplasmic or cytoplasmic face of the NPC [6], suggesting that this asymmetry is important for certain aspects of NPC function, despite being apparently dispensable for the basic mechanisms of transport [13].

Ions, metabolites, and proteins <40 kDa can freely diffuse through the NPC between the cytoplasm and nucleoplasm [14,15]. Larger cargos require nuclear localization signals or nuclear export sequences to mediate transport through the NPC, via interactions with soluble transport factors or karyopherins, which themselves interact with the FG-Nups [16,17]. Directionality is provided by a RanGTP/RanGDP gradient, with RanGTP the predominant form in the nucleus and RanGDP in the cytoplasm reviewed in [18]. However, bulk mRNA export is an ATP-dependent and Ran-independent process, unlike protein transport, with directionality provided by a DEAD-box ATP helicase attached to the conserved cytoplasmic Nup82 (yeast) or Nup88 (vertebrates) complex, which remodels ribonucleoproteins (mRNPs) as they exit the nucleus [19–25].

**Fig 1:**
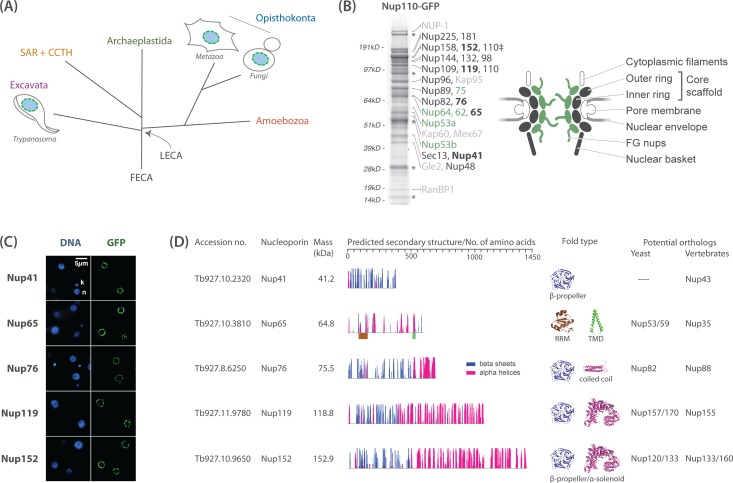
Affinity capture of the trypanosome NPC and identification of new Nups. (A) Schematic of the eukaryotic phylogenetic tree, adapted from Field et al., 2014 [38], highlighting the close evolutionary distance between yeast and humans versus more divergent eukaryotes such as trypanosomes (Excavates). SAR and CCTH correspond to Stramenoplies, Apicomplexa, Rhizaria and Cryptophyta, Centrophelida, Telonimia, Haptophyta, respectively. FECA and LECA refer to the first and last eukaryotic common ancestors. (B) Using the green fluorescent protein (GFP)-tagged nuclear basket protein Nup110 (marked with a ‡), we affinity isolated structural components of the NPC (dark grey), FG repeat containing Nups (green), and specifically associated proteins (light grey), which include transport factors and the major trypanosome lamina protein NUP-1. Affinity isolates were resolved by SDS-PAGE and visualized by Coomassie staining. Protein bands were excised and identified by mass spectrometry (MS). We discovered five new nucleoporins (in bold); assignments are based on secondary structure prediction and localization, as well as multiple pullouts that indicate bona fide association with trypanosome NPC components. Putative nuclear envelope proteins, α/β tubulin, and known contaminants (immunoglobulin heavy chain, variant V_HH,_ and light chains of polyclonal llama anti-GFP antibodies) are marked by asterisks. A comprehensive list of all proteins identified is shown in S1 Fig. A schematic of the NPC is shown to highlight the architecture of the NPC, based on the *Saccharomyces cerevisiae* quaternary structure. Grey and green shapes represent core scaffold Nups and FG-Nups, respectively, identified by DeGrasse et al., 2009 [27]. White shapes represent subcomplexes for which components were not identified in that earlier proteomic screen. (C) Direct visualization of the GFP-tagged newly identified Nups confirms that they exhibit the punctate nuclear rim localization characteristic of NPCs. The corresponding 4’, 6-diamino-2-phenylindoledihidrochloride (DAPI) fluorescence was used to image the DNA (k = kinetoplast, n = nucleus). (D) Secondary structure features and fold prediction of the five newly identified Nups. The *y*-axis indicates the confidence score of the predicted secondary structure element. Models of fold types are shown on the right, together with potential opisthokont orthologs based on the predicted fold types. RRM, RNA recognition motif; TM, *trans*-membrane domain. Fold models are based on PDB structures: 1XIP (β-propeller of Nup159), 3P3D (RRM of Nup35), 2KA2 (TM), 1AQ5 (coiled coil), and 4MHC (α-solenoid of Nup192). TbNup152 is approximately 153 kDa but has been assigned 152 to prevent confusion with the well-studied human Nup153.

Our current understanding of how nucleocytoplasmic transport works stems from decades of work in yeast and vertebrates, both members of the Opisthokonta, one of five or six major supergroups of the eukaryotic lineage (Fig 1A) [26]. NPC components have been catalogued for yeast, several vertebrates, the plant *Arabidopsis thaliana* (Archaeplastida) [7,8], and the trypanosome *Trypanosoma brucei* (Excavata), by us [27]. There is remarkably low sequence similarity between trypanosome and opisthokont Nups, with only five being easily identifiable by sequence alignments [27]. Despite this low sequence similarity, trypanosome NPC components share, to a remarkable level, domain organization and composition with opisthokont Nups. This suggests that most components of the NPC are evolutionarily conserved, albeit with a few exceptions, including the metazoan-specific Nup358 (Ran-binding protein 2), compositional variation in Poms, and the presence or absence of two or three β-propeller proteins in the outer ring of the core scaffold, together with the duplications of Nups in yeast, such as Nup157/170, Nup53/59, or Nup100/116/145N as homologs of the vertebrate Nup155, Nup35, and Nup98, respectively [5,6,27–29]. Indeed, although comparative genomics does not allow full reconstruction of NPC composition for most taxa, data are consistent with overall broad conservation [30]. However, only the yeast NPC has been comprehensively characterized to the architectural level, with partial characterization for vertebrates [3,4,31]. There is, therefore, a significant gap in our understanding of NPC structure and function, as accumulating data suggests significant architectural divergence between different taxa. For example, each vertebrate outer ring is comprised of two reticulated rings, but is a single ring in yeast [4,31,32]. Interestingly, there is a major role for Nup358 in the formation and maintenance of the reticulated cytoplasmic outer ring in metazoa [33]. In trypanosomes, both major components of the nuclear basket, TbNup92 and TbNup110, are highly divergent from the analogous proteins of plants, yeast, vertebrates, and flies [34–37].

We have previously identified and green fluorescent protein (GFP)-tagged 22 *T*. *brucei* Nups (TbNups), to which we assigned putative yeast and human orthologs based on secondary structure prediction and molecular weight [27,39]. Opisthokont and plant NPCs contain about 30 proteins [5–7], suggesting that several TbNups had yet to be identified, and so the absence of a complete NPC composition precluded functional predictions. Further, the arrangement of subunits was unknown. Affinity capture/mass spectrometry (MS) interactomics directly addresses these issues by providing high-resolution mapping and exhaustive analysis of quaternary structure and subunit composition. With this strategy, combined with fluorescence and immunoelectron microscopy (iEM), we have characterized the architecture of the trypanosome NPC, uncovering distinct architectural features that provide novel insights into the function and evolution of this central component of eukaryotic cells.

## Results

### A Strategy to Map Trypanosome NPC Quaternary Structure

**Fig 2:**
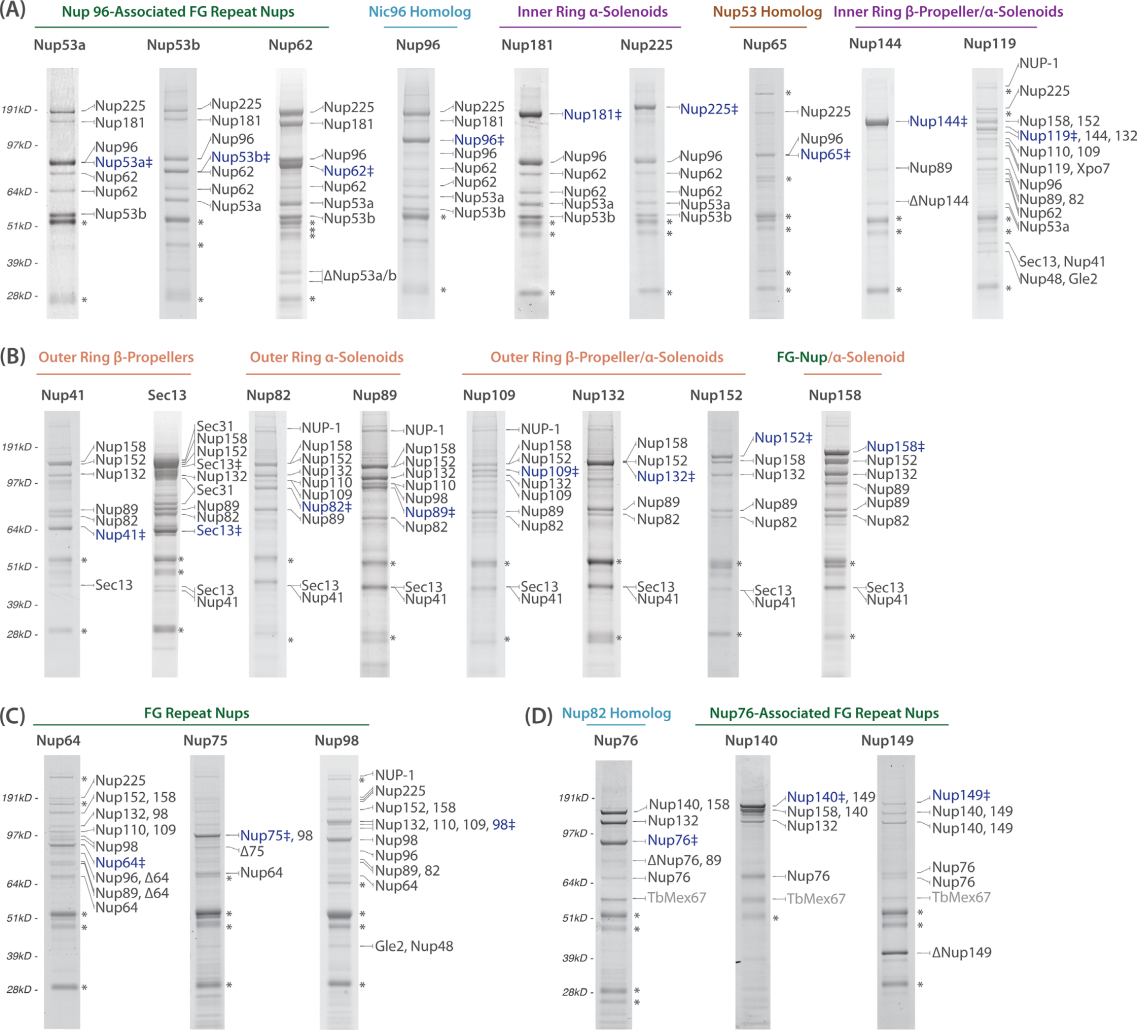
Affinity isolation of TbNPC subcomplexes. TbNup nomenclature has been shortened to NupX, with subsequent comigrating Nups simply given their identification number that corresponds to their molecular weight, with the exception of Sec13 (i.e, Nup158, 152 instead of TbNup158, TbNup152). (A) Coomassie-stained SDS-PAGE of GFP-tagged members of the inner ring of the TbNPC. Predicted homologs, predicted fold types, and the GFP-tagged Nup are shown above each gel. The affinity handle (blue ‡) and isolated proteins identified by mass spectrometry are shown on the right of each protein gel. The asterisks designate known contaminants and non-NPC/nuclear envelope proteins as indicated in Fig 1. Full lists are available in S1 Fig. Nup225, 181, 96, 62, 53a, and 53b form a distinct complex with each other. Nup62 exists as two proteins of different sizes that probably reflect allelic variation due to expansion or contraction of FG-repeats. Nup65 associates with Nup96 and 225. Nup144 weakly interacts with Nup89, whilst Nup119 associates with multiple nuclear pore subcomplexes. (B) Affinity isolated members of the outer ring of the TbNPC. Most of the Nups associate with each other, with a few minor exceptions. Nup109 associates weakly with the rest of the complex and is lost in most affinity capture conditions. However, it is a bona fide member of the outer ring, as it affinity isolates the corresponding members of the Nup89 complex. The Nup89 complex also interacts with the lamin analog NUP-1 [42], the nuclear basket Nup110 [27,35], and the FG-Nup98. The presence of Sec13 in both the NPC and COPII complex is highlighted by the affinity capture of Nups as well as the abundant Sec31, a vesicle coat protein that forms a heterotetramer with Sec13 [43], when Sec13-GFP is used as the affinity handle. (C) FG-Nup64 and 98 associate with multiple NPC subcomplexes. Nup75 only interacts with Nup64 and 98, suggesting a close association of these three FG-Nups. (D) Affinity isolation of Nup76 and several FG-Nups with their interacting partners. Nup76 associates with FG-Nups 140, 149, and several members of the outer ring complex. Additionally, the mRNA export factor Mex67 associates with this subcomplex.

**Fig 3:**
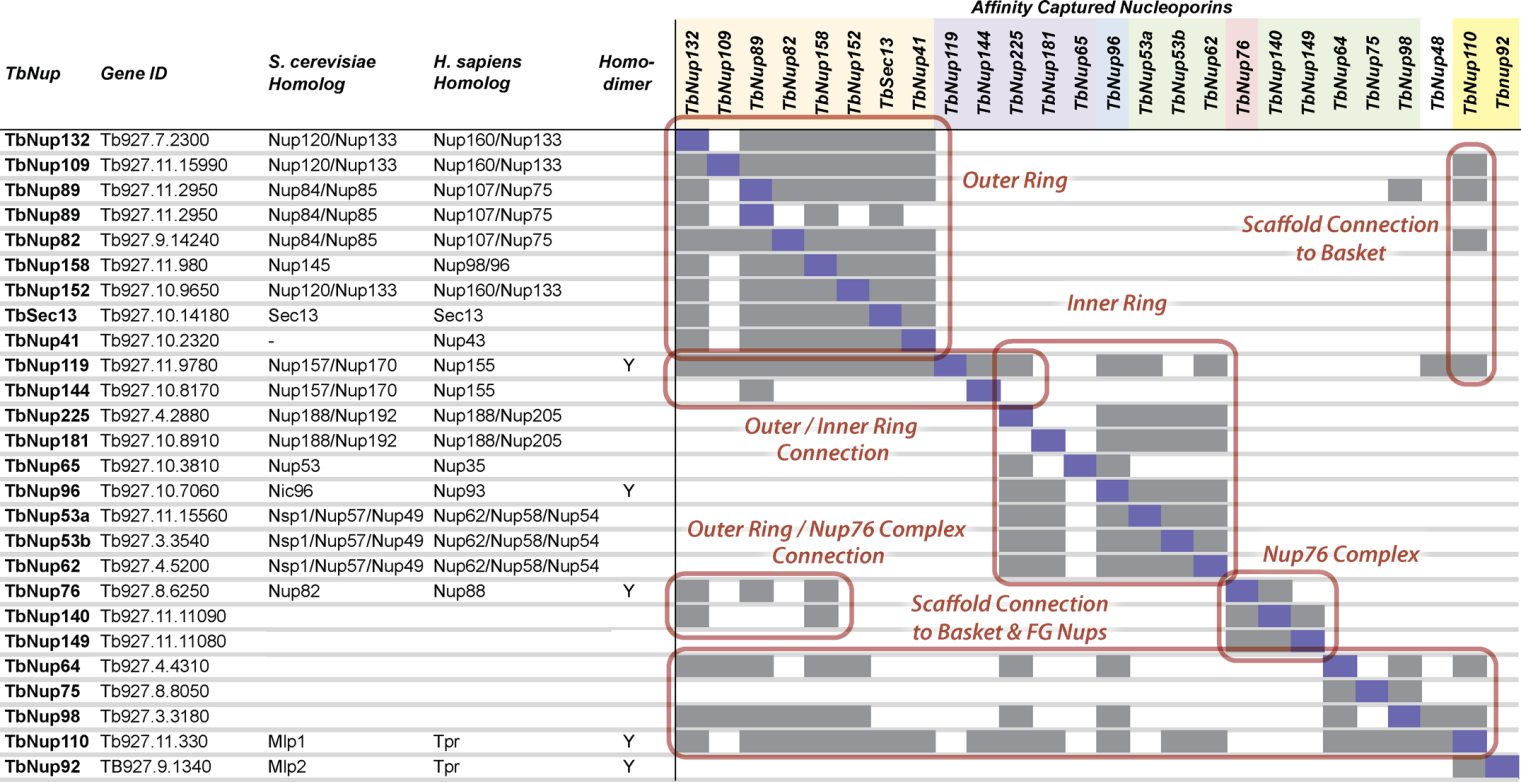
Summary of affinity capture of all Nups. Affinity capture data in Fig 2 are summarized in the above figure and delineate the discrete subcomplexes and the connections between them that define TbNPC subcomplexes and higher order architecture. TbNups that form homodimers are noted, as are the putative yeast and human orthologs of each Nup. The peach color on the label represents outer ring Nups. Purple = inner ring α-solenoids and β-α Nups, blue and pink represent the linker Nups, green = FG-Nups, yellow = nuclear basket Nups, and white = TbNup48/ALADIN, which was not characterized in this study due to our inability to find co-isolating Nups, despite testing several affinity isolation conditions.

**Fig 4:**
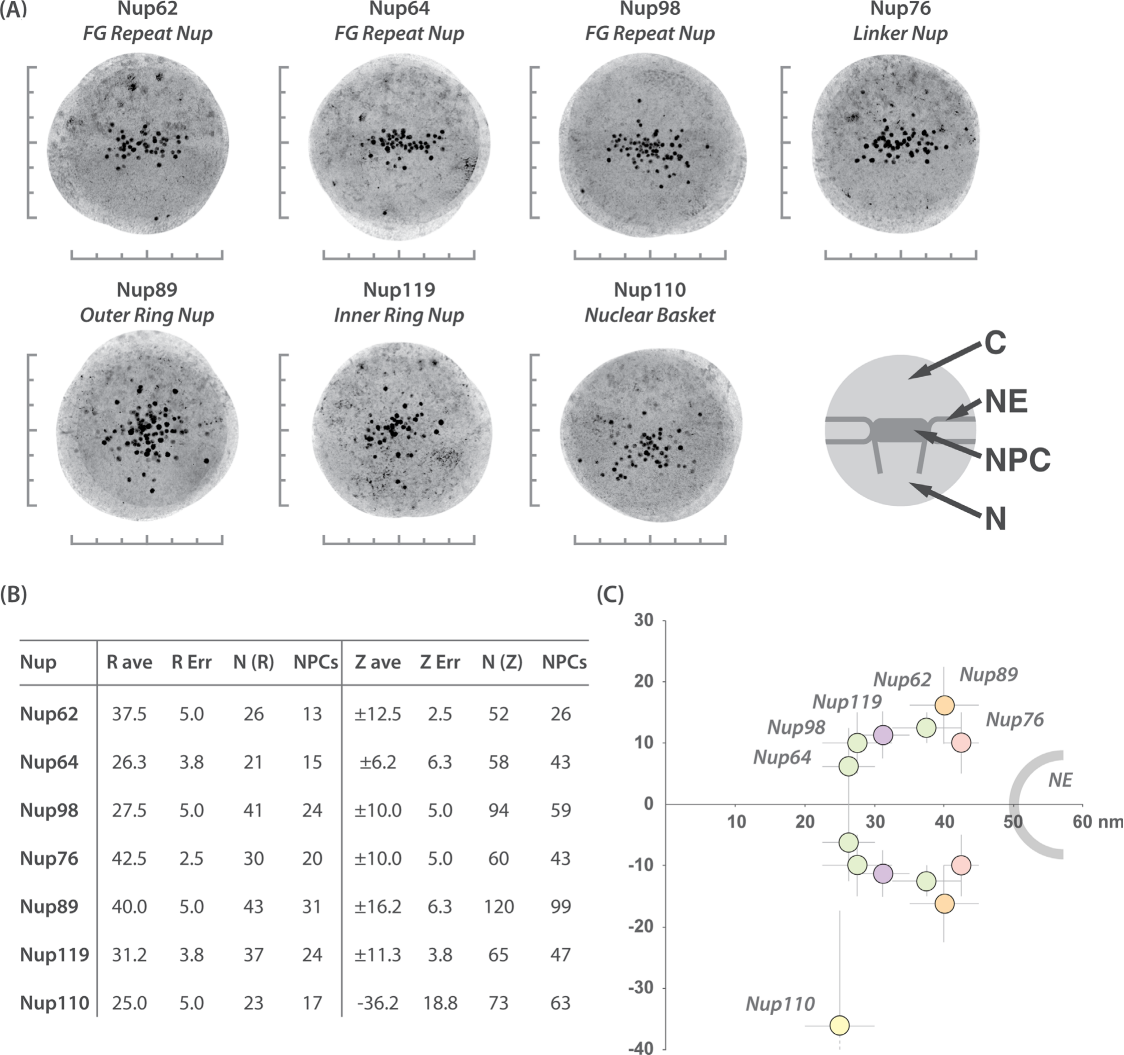
Determination of the relative NPC location of each subcomplex. (A) Immunogold electron localization of GFP-tagged Nups using polyclonal anti-GFP rabbit antibodies to determine relative positions of Nups within the TbNPC (Methods). We picked NPCs sectioned perpendicular to the NE plane, selected a radius of 300 nm around the estimated center of each NPC, and excised each image (S2 Fig). We then aligned and created a superimposed montage of several excised NPC images [6,58]. Graduated lines adjacent to each iEM montage are scaled to represent distances of 50 nm. Major features of each montage are represented in the illustration on the right: NE, nuclear envelope; NPC, nuclear pore complex; N, nucleoplasm; and C, cytoplasm. (B) Statistical analysis of relative locations of select TbNups within the TbNPC, based on the distribution of gold particles from various iEM montages. X and Y positions of gold particles from iEM montages for each selected Nup were measured, from which the Z- and R- (cylindrical rotational axis of the NPC) axes were calculated and displayed in a tabulated form (see S3 Fig, S1 File, and the full table in S2 Table). Z average values are positive or negative to represent localizations above and below the midplane of the NPC. TbNup110 only has a negative value, as it clearly localizes to the nucleoplasm only. Abbreviations: ave (average), Err (error), N(R) (number of gold particles used to calculate the R-axis), N(Z) (number of gold particles used to calculate the Z-axis), NPCs (number of NPCs used to generate either the N(R) or N(Z) for each selected TbNup). (C) Illustrated representation of the relative position of each Nup within the TbNPC. Nup64, 98, and Nup119 are centrally located, whereas Nup62, 76, and 89 appear to be positioned further away from the central channel. The nuclear basket TbNup110 has a clear nucleoplasmic localization. R- and Z-axes errors are plotted based on the 95% level of a peak finding algorithm [6].

Each described trypanosome Nup was tagged in situ at one allele with GFP [27]. All transgenic parasite lines continued to proliferate normally, indicating that the tag has little impact on cell viability and, likely, NPC function. Tagged cells were expanded, rapidly frozen, and then cryomilled (Methods) [40]. The frozen powder was thawed into various buffers to determine optimum conditions for the isolation of the GFP-tagged Nup together with associating proteins. Complexes were captured using polyclonal anti-GFP antibodies conjugated to magnetic beads. Systematic testing of buffers, detergents, salts, and co-solvents allowed us to affinity purify stable NPC subcomplexes, to preserve interactions between NPC subcomplexes, and also to isolate the entire NPC (Figs 1, 2 and 3; S1 Table; see Fig 3 for a comparison of trypanosome and yeast/human Nup orthologs) [41]. By iteratively repeating these affinity capture purifications, we were able to “walk through” the NPC, robustly characterizing a comprehensive NPC interactome, and ensuring that as full a complement of trypanosome Nups as possible was retrieved. Any new candidate component of the NPC was GFP-tagged, had its location confirmed by fluorescence microscopy, and was subsequently used as an affinity handle for further affinity capture experiments (Figs 1 and 2). We also performed iEM on key members of each subcomplex, including FG-Nups that form interactions with multiple subcomplexes (Fig 4). This has allowed us to map the architecture of the trypanosome NPC.

### Using the Nuclear Basket to Uncover the Full Complement of Trypanosome Nups and NPC-Associated Proteins

Previous interactomic analyses in yeast used the nuclear basket components as affinity handles under low stringency conditions to capture essentially the entire NPC [44,45]. We therefore used the nuclear basket component TbNup110 [27,35] as an affinity handle to similarly uncover as full a complement of TbNups as possible. As expected, affinity isolation of TbNup110 under low stringency conditions demonstrated extensive interactions with most of the NPC, recovering most known subunits (Figs 1A and 2) [27,35]. Importantly, we recovered five new nucleoporins, designated TbNup41, TbNup65, TbNup76, TbNup119, and TbNup152 (Fig 1A–1C), which were present in our earlier nuclear envelope proteome, but of insufficient sequence similarity to Nups to warrant inclusion in that study [27]. No additional bona fide TbNups were identified from either these or any of our extensive affinity capture experiments. However, we did isolate the lamina protein NUP-1 [42] and several candidate NE proteins, indicating that the procedure has likely saturated identification of NPC components and, indeed, reached beyond it. These data provide robust confirmation that we have likely identified the full complement of trypanosome Nups (see S1 Fig for complete list of identified proteins).

### The Inner Ring Is an Ancient and Highly Conserved Core NPC Structure

We first wanted to ask: are the main architectural features that have been defined in opisthokont (yeast and vertebrate) NPCs also conserved in the trypanosome NPC? In opisthokonts, among the most conserved such features are the inner ring of the NPC (Fig 1), which in yeast is comprised of two large α-solenoid proteins, ScNup192 and ScNup188, and two β-α structured paralogs, ScNup157 and ScNup170 [9,46–50]. These four proteins interact with the membrane ring that anchors the NPC to the pore membrane, as well as to the ScNic96 complex [4,50,51]. ScNic96 is an evolutionarily conserved and highly abundant α-solenoid protein, which itself is in a complex with three central channel FG-Nups (ScNup57, 49, and Nsp1) in yeast [52]. This entire inner ring arrangement appears very similar in vertebrates [53,54].

TbNup96 can be readily identified as orthologous to ScNic96 in silico, establishing that this protein is conserved. However, sequence comparisons alone do not fully discern the level of conservation of any other putative inner ring components, or, indeed, if there is an inner ring [27]. Thus, we used affinity capture of TbNup96 in order to “walk out” from this protein to uncover its molecular neighborhood. Affinity isolation of TbNup96 co-purified the two largest α-solenoid proteins in the *T*. *brucei* NPC (TbNPC)—TbNup225 and TbNup181—as well as three FG-Nups: TbNup62, TbNup53a, and TbNup53b (Figs 2A and 3). Reciprocal affinity isolates of TbNups62, 53a, and 53b co-purified both TbNups225 and 181 as well as each other (Fig 2A). Interestingly, affinity isolation of TbNup225 co-purified all members of the complex except TbNup181 (Fig 2A). Likewise, affinity isolation of TbNup181 co-purified TbNups96, 62, 53a, and 53b, but not TbNup225 (Fig 2A). These data suggest that TbNup225 and TbNup181 do not interact directly, but rather form two distinct subcomplexes, each containing TbNup96, 62, 53a, and 53b; this is further supported by the affinity capture of Nup96, which co-purifies with both TbNups181 and 225 as well as an untagged form. A similar two-complex architecture is present in two fungi, *Saccharomyces cerevisiae* and *Chaetomium thermophilum* [4,55]; in *C*. *thermophilum*, the orthologous CtNup192 and CtNup188 compete for the same 90 amino acid binding site on an α-helical motif near the N-terminal of Nic96. The vertebrate orthologs of the Nic96 complex appear similarly organized [56]. Thus, the association of the two large α-solenoid proteins with TbNup96 and three FG-Nups indicates that the composition of this complex represents an extremely conserved module (Fig 3), also definitively assigning TbNup62, 53a, and 53b as orthologs of ScNup57, 49, and Nsp1, with which they share clear domain similarities.

TbNup144 and 119 are composed of an N-terminal β-propeller and a C-terminal α-solenoid (β-α) (Figs 1C and 2A) [27]. TbNup144 is evolutionarily well conserved and orthologous to ScNup157/170 and HsNup155 [27]. In contrast, TbNup119 has weak sequence similarity to ScNup170, based on secondary structure prediction modeling with Phyre2 (www.sbg.bio.ic.ac.uk/phyre2/) [57]. Affinity isolation of TbNup144 reveals an interaction with only the α-solenoid TbNup89 (Fig 2A), whereas TbNup119 co-purified with a large number of TbNups (Figs 2A and 3); thus, it appears that TbNup144 links to the outer ring (see below) through interactions with TbNup89, whilst TbNup119 has extensive connections with the core scaffold of the TbNPC (Fig 3).

We performed post embedding (in resin) iEM gold labeling of the NPC using selected GFP-tagged TbNups as described by Krull et al., 2004 (Figs 4, S2 and S3) [58]. The advantage of post resin embedded labeling on whole cells is superior preservation of NPCs, as they are within their correct cellular environment with no manipulation other than high-pressure freezing. Additionally, there is the benefit of being able to label both externally and internally localized GFP-tagged Nups within the context of the NPC. However, good preservation comes at the expense of signal; because the antigens are embedded in plastic resin, only GFP epitopes exposed on the resin surface are accessible for labeling [59–62].

Using the resulting gold particle distributions, we used our prior methods [3,4] and those of Krull et al., 2004 [58] to provide a preliminary estimate for the position of each protein in the NPC (Methods). Consistent with being a conserved central channel FG-Nup, TbNup62 has a symmetric distribution centered tightly around the median plane of the TbNPC, adjacent to the putative central channel. TbNup119 displayed a very similar distribution to TbNup62, consistent with its assignment as another component of the inner ring core scaffold. By contrast, and in confirmation of the relative accuracy of our iEM methodology, the nuclear basket component TbNup110 has a nucleoplasmic localization centered fully ~40 nm from the median plane of the TbNPC (Fig 4). Taken together, these data strongly support that the entire inner ring structure and composition is highly conserved across the eukaryotes.

### Plasticity in Membrane Anchoring Mechanisms

**Fig 5:**
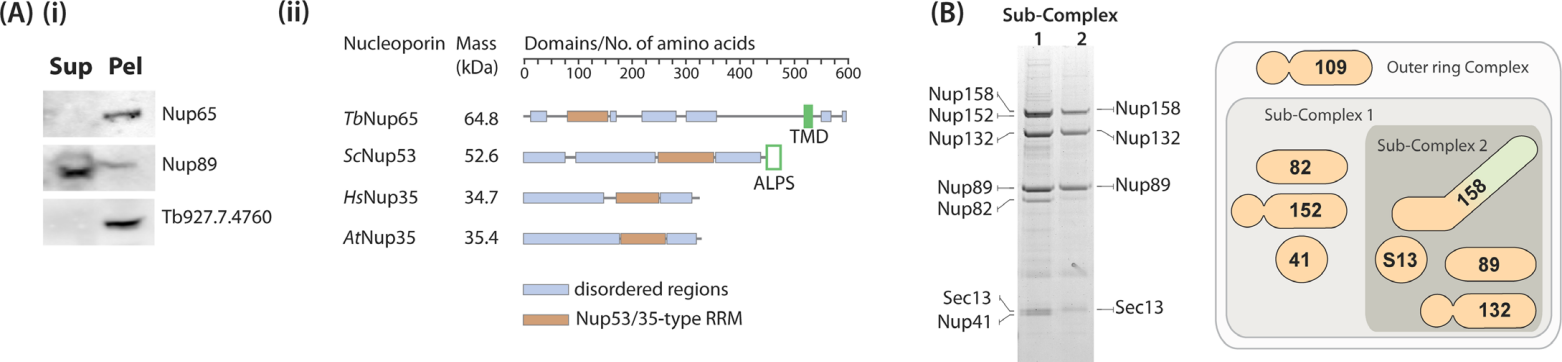
Membrane anchoring and the core module of the TbNup89 complex. (A) TbNup65 is a TM containing protein. (i) Western blot showing sodium carbonate extraction of TM proteins [64], confirming that TbNup65 and Tb927.4.4760—a nuclear envelope and Golgi marker protein—are TM proteins, as they are predominantly recovered in the pellet (Pel) whilst the non-TM α-solenoid TbNup89 is predominantly recovered in the supernatant (Sup). (ii) An illustration of the predicted secondary structure and the differences in nuclear membrane interaction between TbNup65 and its yeast, human, and plant orthologs (ScNup53, HsNup35, and AtNup35, respectively). The opisthokont and plant Nup53/35 are mainly disordered (Disopred), unlike the trypanosome Nup65 that has several structured regions. (B) The Nup89 complex is comprised of eight proteins (including TbNup109) that can be further reduced into a core module consisting of just four proteins when the stringency of the extraction buffer is increased. A schematic of the outer ring as well as subcomplexes is shown. Nup41 and Sec13 are beta propellers, Nup82 and 89 are alpha solenoids, Nup109, 132 and 152 are beta/alphas and Nup158 is a FG-Nup/alpha solenoid.

The NE is an invariant feature of NPCs and, as such, one would imagine that the membrane anchoring structures of the NPC would be very highly conserved. Remarkably, however, there appears to be an absence of any identifiable orthologs of the opisthokont *trans*-membrane anchoring Poms (ScPom152/HsGp210, ScNdc1/HsNdc1, ScPom34, and HsPom121) in the trypanosome NPC interactome. Nonetheless, we identified a Nup with a TM domain, but which was intriguingly different from those in opisthokonts. TbNup65, a newly identified TbNup, appears orthologous to ScNup53/59 and HsNup35 (Fig 1) and contains an RRM (RNA recognition motif) domain also found in these opisthokont proteins [63] at residues 81– 153 (Figs 1C and 5A). The Nup35-type RRM is a noncanonical ribonucleoprotein motif that lacks key residues involved in RNA binding, making it identifiable by bioinformatics [63]. However, the most intriguing feature of TbNup65 is the presence of a predicted TM domain at residues 516–535 (Fig 5A). This TM domain is present in all kinetoplastid Nup65 homologs (S4 Fig). The presence of a TM domain in TbNup65 was confirmed by carbonate extraction, where TbNup65 was recovered exclusively in the pellet, behaving identically to another predicted TM protein, Tb927.7.4760, which localizes to both the nuclear rim and the Golgi (S5 Fig). This is distinct from TbNup89, which possess no predicted TM domain and, as expected, was efficiently extracted by carbonate (Fig 5A).

In opisthokonts, ScNup53/HsNup35 connect the pore membrane to the core scaffold of the NPC, a role critical for assembly [65,66]. However, the interaction of ScNup53/59 with the pore membrane is mediated by an amphipathic lipid-packing sensor (ALPS) motif at the C-terminus of each protein, and which associates with membranes [67–69]. Significantly, the ALPS motif and TM domains use different mechanisms of membrane association, as the former does not traverse the membrane [69,70]. TbNup65 interacts with TbNup96 and TbNup225 (Fig 2A), interactions that are conserved with the respective yeast and vertebrate orthologs [55,65,69,71,72]. In yeast, ScNup53 interacts directly with inner ring ScNup170 [4,73]. In vertebrates, Nup35 (ScNup53) interacts with inner ring proteins Nup93 (ScNic96), Nup155 (ScNup157/170), Nup205 (ScNup192), and the pore membrane protein NDC1 [65,71,72]. Thus, while connections between TbNup65 and the NPC appear largely conserved, the mechanism anchoring the NPC to the pore membrane appears to be distinct, and the moieties used to anchor the NPC to the pore membrane (TM domains, ALPS motifs) are interchangeable.

### The Outer Ring: Conservation and Variation

The next question we addressed was: does the level of conservation found in the inner ring component of the core scaffold extend to the outer ring? The outer rings are located on the cytoplasmic and nucleoplasmic faces of the NPC and are dominated by α-solenoids, β-propellers, or the β-α structure [4,6,10,31,58]. The architecture of both the yeast and vertebrate outer rings are comparatively well characterized, permitting more detailed comparisons.

TbNup158 is a clear ortholog of the yeast outer ring component ScNup145/HsNup98-96 [27]. Affinity capture of TbNup158 recovers six additional TbNups: TbSec13, TbNup41, TbNup82, TbNup89, TbNup132, and TbNup152 (Fig 2B). This septameric complex is repeatedly recovered in multiple affinity captures using these proteins as handles, with an additional protein, TbNup109, recovered in the affinity capture of TbNup82, suggesting these proteins may interact directly. Affinity capture using TbNup109-GFP itself confirms TbNup109 as a bona fide member of the same complex, as it co-purified all members of the complex (Figs 2B and 3) as well as being recovered in the entire TbNPC isolation (Fig 1A), suggesting TbNup109 is an easily displaced component. Under more stringent affinity isolation conditions, we find that the complex can be delimited to a module comprised of TbSec13, TbNup89, TbNup132, and TbNup158 (Fig 5B). Localization of a defining member of this complex by iEM, Nup89, shows that it is both axially and radially more distal from the central channel than the inner ring components (Fig 4), consistent with being part of a TbNPC outer ring. Hence, we named this complex the TbNup89 complex, likely representing the outer ring equivalent of the ScNup84 complex and HsNup107-160 complex [27,32,74–79].

**Table 1:** A comparison of the individual units of the evolutionarily conserved outer ring complex between trypanosomes, opisthokonts, and plants.

Secondary structure	Trypanosomes	Yeast	Vertebrates	Plants
**α-solenoid**	TbNup158	Nup145C	Nup96	Nup96
	TbNup89	Nup85	Nup75	Nup75
	TbNup82	Nup84	Nup107	Nup107
**β-propeller α-solenoid**	TbNup152	Nup120	Nup160	Nup160
	TbNup132	Nup133	Nup133	Nup133
	TbNup109	-	-	-
**β-propeller**	TbSec13	Sec13	Sec13	Sec13
	-	Seh1	Seh1	Seh1
	TbNup41	-	Nup43	Nup43
	-	-	Nup37	-

The outer ring complex is well conserved with species-specific differences revolving around the presence and absence of β-propeller proteins. However, the most significant difference is the presence of an additional β-propeller/α-solenoid Nup in trypanosomes that is clearly missing from other taxa studied so far.

The composition of the TbNup89 complex reveals a high degree of architectural conservation of the outer ring complex across eukaryotic evolution (Table 1). However, there are significant differences highlighted in Table 1. The most prominent is the presence of three β-α Nups, TbNup109, 132, and 152, as opposed to just two in opisthokonts [5,6]. Remaining differences revolve around the presence or absence of the small β-propeller proteins Seh1, Sec13, Nup37, and Nup43. Sec13 is present in all characterized versions of this complex, likely through a direct association with orthologs of TbNup158 [76]. Both Nup37 and Nup43 are absent from the *S*. *cerevisiae* Nup84 complex, but orthologs of Nup37 are present in other fungi, including *Aspergillus nidulans*, *Schizosaccharomyces pombe*, and *C*. *thermophilum* [80–83]. This compositional flexibility is also apparent in the absence of a recognizable Seh1 ortholog in the trypanosome NPC, just as in the NPCs of thermophilic fungi [83,84] and in affinity captures of the TbNup89 complex. Rather, TbNup41, the only other β-propeller protein in the TbNup89 complex besides TbSec13, appears to have a distinct ancestry to that of Seh1, as determined by phylogenetic analysis, and is likely orthologous to Nup43 (S6 Fig). Overall, therefore, the outer ring—though carrying many conserved features—has more lineage-specific subunits than the inner ring.

### A Simpler and More Symmetric Distribution of FG-Nups

The degree of conservation of the peripheral components of the NPC is much less established. Candidate proteins corresponding to components of the cytoplasmic fibrils, and specifically orthologs of the linker Nup ScNup82/HsNup88 or FG-Nups ScNup159/HsNup214 and ScNup42/hCG1 that are crucial for mRNA export, have never been identified in trypanosomes [6,27,85–93]. Likewise, no apparent orthologs of the nuclear-face-localized FG-Nups ScNup1 or ScNup60 have been identified in trypanosomes [6,27].

We can now assign several trypanosome FG-Nups to specific locations within the NPC, depending on the scaffold Nups with which they stably interact and co-purify. As described above, TbNup53a, TbNup53b, TbNup62, and TbNup158 are all symmetrically disposed FG-Nups, facing both the cytoplasmic and nucleoplasmic faces on the NPC (Figs 2 and 3). However, we could not accurately determine the localization of TbNup64, TbNup75, and TbNup98 by affinity capture alone, as they interact with both inner and outer ring scaffold Nups as well as the nuclear basket (Figs 2C and 3). The FG-Nups TbNups64 and 75 are paralogs, with near-identical amino acid sequences, albeit with several insertions in TbNup75 that are responsible for the size difference between the two. We presume TbNup75 function and localization to be similar to TbNup64, as they interact directly (Fig 2C). To more accurately determine the sublocalization of these FG-Nups, we performed post embedding iEM gold labeling for TbNup64 and TbNup98 (Fig 4). We found that both have a symmetric distribution in the trypanosome NPC, close to the central channel and the NPC’s equator, consistent with their strong interactions with both the inner and outer ring.

Affinity capture of the TbNPC identified TbNup76, a predicted β-propeller protein with a short coiled-coil C-terminal region (Fig 1). This secondary structure is similar to that of ScNup82/HsNup88, the only opisthokont or plant Nup (AtNup88) with this architecture, suggesting that they are orthologs (Fig 1C) [7–9]. This orthology is supported by the observation that capture of tagged Nup76 also yields an untagged copy of itself (Fig 2D), suggestive of the same kind of dimeric architecture found in opisthokonts [4,94].

Affinity isolation of TbNup76-GFP identifies it as part of an NPC subcomplex containing the two largest FG-Nups, TbNup140 and TbNup149 in the TbNPC, which also co-purify with each other and Nup76 (Figs 2D and 3). This complex interacts with some members of the TbNup89 complex, specifically TbNup132 and TbNup158 (Fig 3). The interaction between TbNup76 and the TbNup89 complex suggests that the latter may anchor TbNup76 and its associated FG-Nups. High-density FG repeats (101 in total) comprise 117 kDa of TbNup140, while the N-terminal region contains a 23 kDa predicted coiled-coil [27]. By contrast, TbNup149 is not as FG-rich (18 FGs) and is composed of three near identical repeated domains that comprise the entire protein (S7 Fig). Additionally, the repeated units have putative zinc finger domains, the significance of which is currently under investigation (S7 Fig). Notably, neither TbNup140 nor TbNup149 has structural similarity to either ScNup159, which has an N-terminal β–propeller domain, or ScNup42, the two cytoplasmic FG-Nups of the yeast NPC (or their vertebrate orthologs), suggesting that the organization of the FG-Nups in trypanosomes is likely distinct.

To directly address this, we localized TbNup76, again using post embedding gold labeling iEM (Fig 4). Surprisingly, TbNup76, the putative ortholog of the cytoplasmically facing Nup82 in yeast, exhibits a symmetric localization, suggesting that it is found on both nucleoplasmic and cytoplasmic faces of the trypanosome NPC. By extension, the FG-Nups TbNups140 and 149, which interact with TbNup76, are predicted to localize symmetrically. Together with the apparently symmetric localization of the other Nups tested by iEM, this unexpected result suggests that the only definitively asymmetrically localized components are the nuclear basket proteins TbNup110 and TbNup92 [27,35], while all other components are equally disposed on the nuclear and cytoplasmic halves of the TbNPC. This is highly distinct from opisthokont NPCs, over a quarter of whose Nups are asymmetrically localized to only their nuclear or cytoplasmic faces. This large-scale architectural difference is likely connected to the absence of obvious orthologs of cytoplasmic or nucleoplasmic-biased FG-Nups, i.e., ScNup159/HsNup214 and HsNup153/ScNup1-Nup60.

### A Divergent Mechanism for mRNA Export

An absence of clear nucleocytoplasmic asymmetry in the trypanosome NPC is remarkable, especially as NPC asymmetry is crucial for driving opisthokont mRNA export [22,89,95]. In particular, the ATP-dependent DEAD box RNA helicase Dbp5 and the RNA export mediator Gle1, with its cofactor IP_6_ (inositol hexakisphosphate), associate with the N-terminal β-propeller of cytoplasmic FG-Nup ScNup159/HsNup214, a member of the ScNup82 complex and remodel messenger ribonucleoproteins (mRNPs) exiting the nucleus [19,20,23–25,96–98]. This allows the non-karyopherin RNA export factors (Mex67:Mtr2 in yeast, TAP:p15 in humans) to disengage and recycle back into the nucleus, providing the necessary directionality and energy to RNA export [99–101]. As well as lacking a ScNup159/HsNup214 ortholog, orthologs of Gle1 and Dbp5 are absent from affinity-captured complexes and cannot be identified in the trypanosome genome. (See S8 Fig for phylogenetic analysis. Files are viewable using the free “Archaeopteryx” software.) By contrast, orthologs of other RNA export factors, including ScMex67:Mtr2/HsTAP:p15 and ScGle2/HsRae1, can be readily identified in trypanosomes [27,102,103].

**Fig 6:**
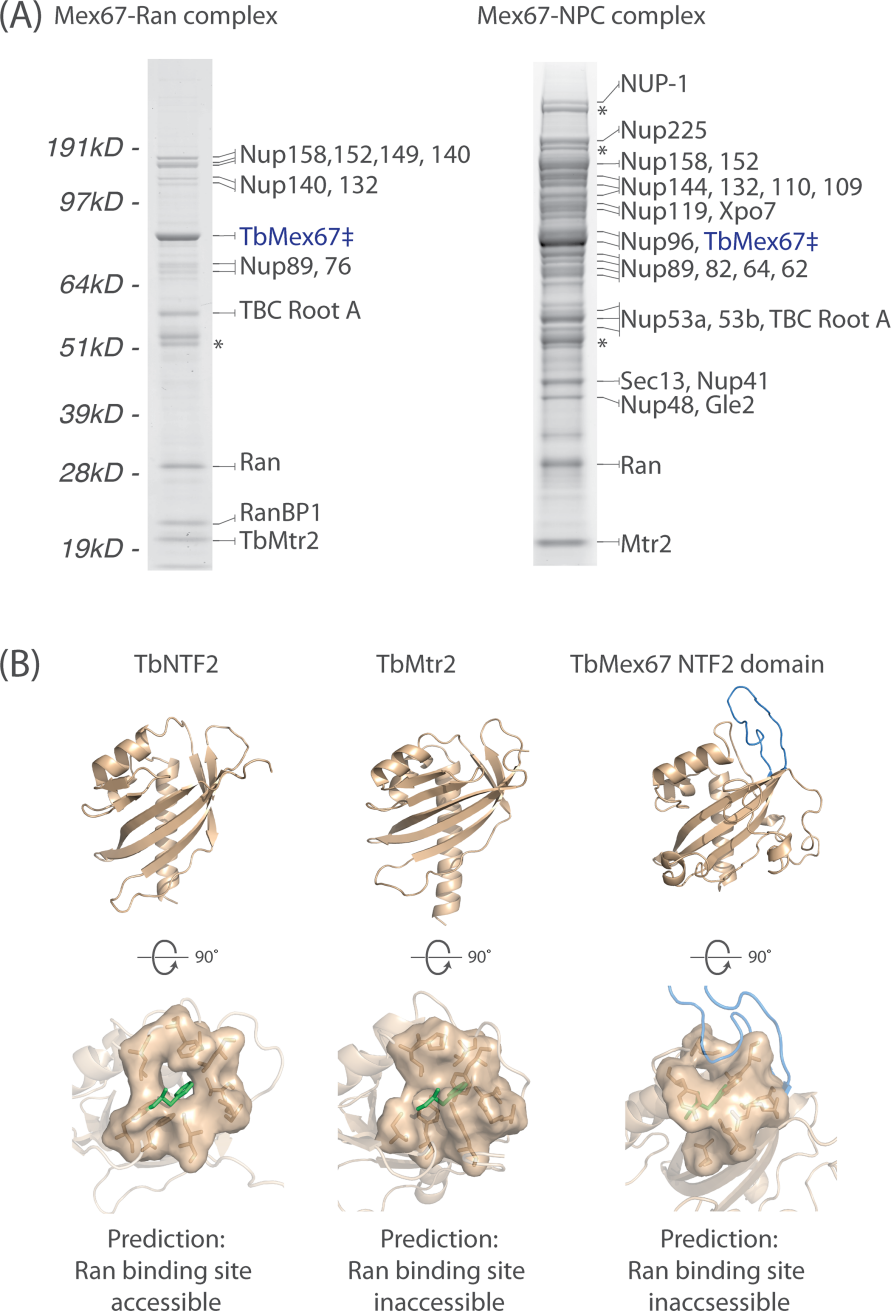
The interactions of an evolutionarily conserved mRNA exporter with the TbNPC and Ran. (A) TbMex67 interacts primarily with the Nup76 complex and several components of the TbNup89 complex. TbMex67 also interacts with Ran, Ran Binding Protein 1 (RanBP1), and a GTPase activating protein (TbTBC-RootA) [104] that shows similarity to Rab GTPase Activating Proteins (GAPs) by protein domain prediction. These interactions are suggestive of a role for the Ran gradient in the export of bulk polyA mRNA export. Under low stringency conditions, the interaction between TbMex67 and the TbNPC is clearly observed in a manner reminiscent to that of yeast Mex67 [40]. (B) Models of the trypanosome NTF2, TbMtr2, and the NTF2 domain of TbMex67 were generated using I-TASSER, resulting in C-scores of 1.14, -0.96, and 0.95, respectively. The C-score is used to assess the quality of a model generated by I-TASSER [105]. Its calculation is based on the Z-score of individual threading alignments and the convergence parameters of the I-TASSER assembly simulations. C-scores range between -5 and 2; the closer the score to 2, the higher the confidence in the model generated. The C-scores generated for our models are closer to 2, reflecting high confidence in the models generated. TbNTF2 is capable of binding Ran, based on an accessible potential Ran-binding pocket [106,107], whereas the potential Ran-binding pocket in TbMtr2 and the NTF2 domain of TbMex67 are predicted to be inaccessible, based on structural modeling using I-TASSER. Significantly, this mirrors the situation in yeast and vertebrates, suggesting that Ran binding may not be direct and probably requires the other Ran interacting proteins such as RanBP1 and TbTBC-RootA.

In opisthokonts, Mex67/Mtr2 interacts with numerous Nups and RNA processing factors, including Gle1 and Dbp5 [40]. Therefore, to understand how Mex67 interacts with the NPC in trypanosomes and assess the composition of any potential RNA processing platform, we affinity captured TbMex67 under a variety of stringencies (Fig 6A). Under high stringency conditions, we found TbMex67 co-isolated with TbNup76, TbNup140, and TbNup149, as well as the highly conserved binding partner of Mex67, TbMtr2 [102]. This strongly implies that the TbNup76 complex is part of the mRNA export factor docking platform. Under low stringency conditions, we co-isolated several TbNups and transport factors. Significantly, no potential orthologs of mRNA export factors Dbp5 and Gle1 were identified (Fig 6A). Thus, the absence of these proteins is suggestive of an mRNA export mechanism that is probably different from that in opisthokonts.

Besides TbNups, TbMex67/TbMtr2 forms a complex with Ran and other putative Ran binding proteins (RanBP1 and GAP TbTBC-RootA). It is unclear whether TbMex67/Mtr2 can bind Ran directly or is doing so via these other proteins (Fig 6A). If direct, presumably the interaction would be via the NTF2-like domains of Mex67 and Mtr2. NTF2 binds to and imports Ran-GDP into the nucleus [107–110]. Ran binds NTF2 via a highly conserved phenylalanine (Phe72), called the “switch II” region, which binds a hydrophobic pocket on NTF2 [107]. In opisthokonts, Ran binding to TAP (Mex67) is blocked by a helix preventing access to the equivalent hydrophobic pocket of the NTF2-like domain in Mex67 [106]. Likewise, Ran binding to opisthokont p15 (Mtr2) is prevented by the presence of large hydrophobic residues in the corresponding hydrophobic pocket that obstruct the incoming Phe72 of the Ran switch II region [106,111]. We were able to generate high confidence models of TbNTF2, the TbMex67 NTF2-like domain, and TbMtr2, because of their sequence similarity to their structurally characterized opisthokont orthologs (Methods). Our models support the binding of NTF2 to Ran in trypanosomes, as the hydrophobic Ran binding pocket in TbNTF2 appears to be accessible and conserved (Fig 6B). Our models also suggest that the Ran binding pocket of the NTF2 domains of TbMex67 and TbMtr2 are occluded and, thus, inaccessible to Ran binding, exactly as in opisthokonts [106]. Thus, based on our models, direct Ran GTP-dependent interaction seems unlikely, rather being through RanBP1 and the GAP (TbTBC-RootA).

## Discussion

How can we reconstruct eukaryogenesis and the pathways that lead to and from the prokaryote/eukaryote transition? One potentially valuable approach is to understand the structures and mechanisms operating at the nuclear envelope from key organisms across the eukaryotic lineage. A detailed comparative dissection of the machinery mediating central functions can enable reconstruction of evolutionary history and origins. The data reported here provide the first comprehensive survey of the architecture of the NPC from a highly divergent organism, providing key insights into evolutionary origins of function and mechanism at the nuclear envelope.

### Analysis of Interactions between Trypanosome Nucleoporins Identifies Conserved and Divergent Components

**Fig 7:**
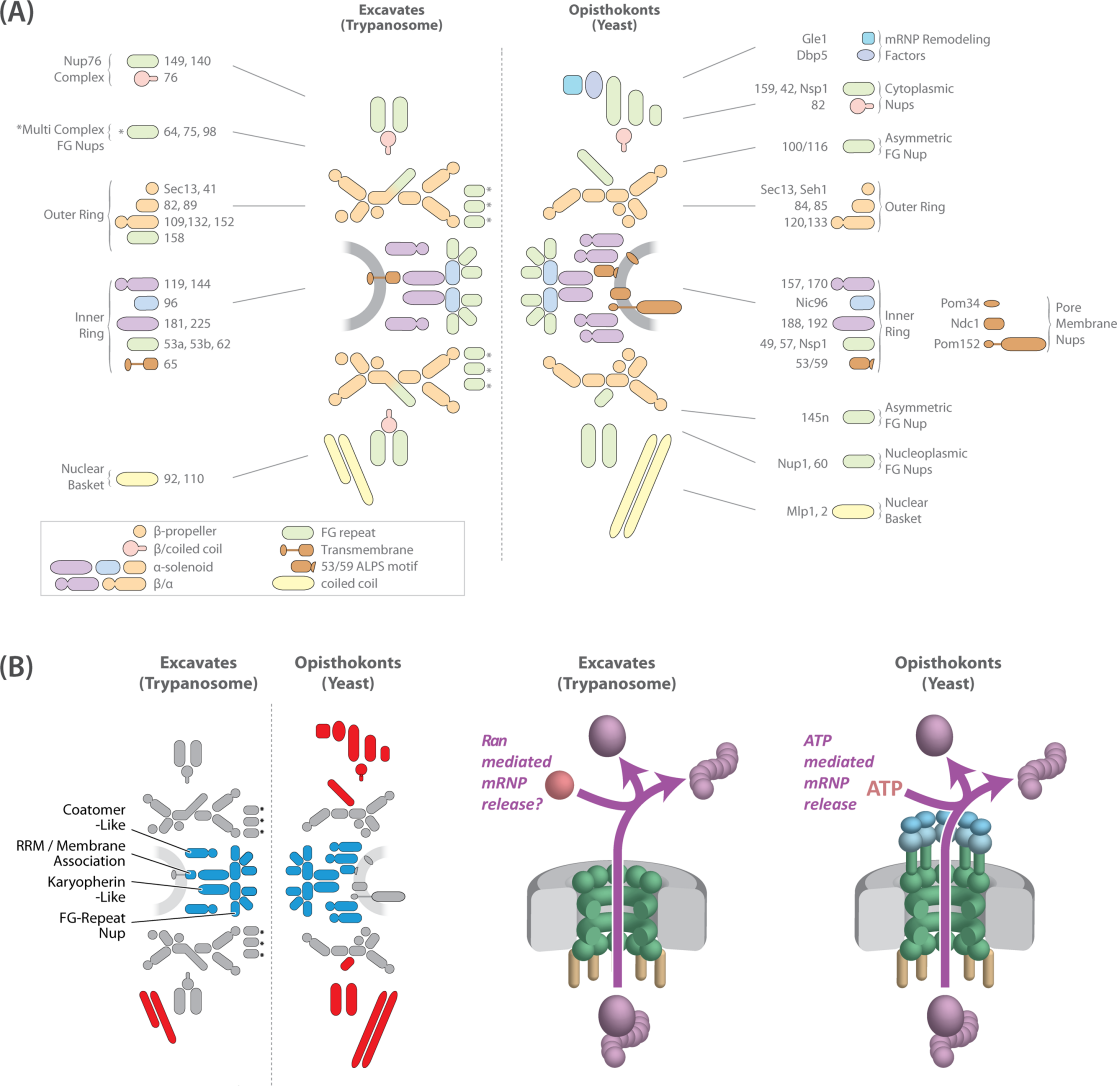
Model of the TbNPC and a putative role of Ran in mRNA export. (A) A model of the TbNPC compared to the yeast NPC. Only one copy of the inner ring is illustrated for simplicity. The anchoring mechanism of the TbNPC is provided by a single inner ring Nup (TbNup65) that in yeast (ScNup53/59) interacts with the NE via an ALPS motif. Trypanosomes lack the whole pore membrane ring comprised of Pom152 (GP210 in humans and plants), Pom34, and NDC1 [5,6]. The TbNPC is largely symmetric, with asymmetry provided by its nucleoplasmic interactions through two nuclear basket Nups that are half the size of their opisthokont analogs [35]. Significantly, there are no clear orthologs of Dbp5 and Gle1, coincident with the lack of cytoplasmic or nucleoplasmic biased FG-Nups in trypanosomes. Instead, TbNup76, the candidate ortholog of the cytoplasm-specific Nup82/88 in opisthokonts, localizes to both faces of the NPC. (B) Left, model highlighting the conserved inner ring core (blue) and differences in asymmetry (red) in excavates and opisthokonts as represented by trypanosomes and yeast. Orthologs of cytoplasmic Nups or mRNA remodeling factors are absent from trypanosomes. Right, affinity capture of the conserved nonkaryopherin RNA exporter Mex67 co-isolates Ran, suggesting a putative role for the GTPase Ran in mRNA export in trypanosomes (see Fig 6A). Bulk polyA mRNA export in opisthokonts is driven by ATP through the actions of the ATP-dependent DEAD box helicase DBP5, RNA export factor Gle1, and inositol hexakisphosphate (IP_6_) [22].

Overall, there is a high degree of conservation between the trypanosome, opisthokont, and vascular plant NPCs at the level of subunit composition, although the trypanosome appears most divergent [5,6,8,27]. Rather than primary structure, conservation is at the level of shared structural domains in similar arrangements. Strikingly, the molecular weights of orthologs are very well conserved (S3 Table) and may reflect severe spatial constraints to assembling a cylindrical structure delimiting a ~40 nm channel, containing correctly spaced gating FG repeats and both spanning and stabilizing the ~50 nm pore membrane. The core scaffold (inner and outer rings) of the NPC, comprised of orthologous proteins carrying coatomer-related α-solenoid, β-propellers, and β-α structures, is highly conserved between trypanosomes, vascular plants, animals, and fungi, but with notable differences (Fig 7A) [5,6,8,10,27]. Significant conservation of this NPC substructure was expected, as it is a member of the protocoatomer group of membrane-deforming complexes that mediate membrane trafficking and intraflagellar assembly and transport (reviewed in [11]). This further evidence supports the paradigm that the ancestor of these membrane-deforming complexes arose via a pre-LECA expansion from an ancestral protocoatomer complex [10,11].

Within the core scaffold, the inner ring is the most conserved component of the NPC, with clear orthologs in vascular plants (Fig 7B) [7,8]. This high degree of conservation was unclear until our survey provided robust evidence that the FG-Nups62, 53a, and 53b were the orthologs of ScNsp1/HsNup62, ScNup57/HsNup58, and ScNup49/HsNup54, respectively, and that the inner ring organization of trypanosome, yeast, and vertebrate NPCs are very similar (Fig 7A). By contrast, the trypanosome outer ring TbNup89 complex displays several divergent features, the most significant of which are the absence of the β-propeller protein Seh1 and the possession of three large β-α structure Nups (Nup152, Nup132, and Nup109) rather than just two, as present in all other lineages examined so far (Table 1). Perhaps three β-α structure Nups are a remnant of an earlier, more LECA-like architecture for this complex, lost in other lineages; further detailed structural mapping of the TbNup89 complex as well as analyses from additional divergent taxa may help resolve this possibility.

### Plasticity in Membrane Attachment

The high level of conservation of inner ring features extends to TbNup65, the ortholog of ScNup53/HsNup35. TbNup65 interacts with the nuclear membrane via an orthodox TM that is conserved between kinetoplastids (S4 Fig) and represents the sole membrane anchor identified in the trypanosome NPC by our methods. That ALPS and TM domains appear functionally interchangeable suggests that the precise mechanism of anchoring the NPC to the nuclear membrane is unimportant, so long as it has some such mechanism (Fig 7A). This idea is supported by the observation that deletion of all TM proteins from *A*. *nidulans* NPCs has no deleterious effects on viability (although the putative ALPS-containing proteins are essential in this context) [81]. The absence of an ortholog to the TM protein ScPom152 in trypanosomes is notable, as orthologs are present in other opisthokonts and plants. Pom152 has a cadherin domain, in common with many membrane receptors and proteins that bridge between two membranes [9]. Thus, while this could reflect lineage-specific loss from trypanosomes, a more attractive interpretation is as an example of neofunctionalisation of a membrane protein into a NPC-specific role, postdating speciation between opisthokonts, plants, and trypanosomes.

### FG-Nups Are Symmetrically Distributed in the Trypanosome NPC

Immunoelectron microscopy localization of several Nups, representing key subcomplexes and Nup classes, showed that all were symmetrically disposed between the nuclear and cytoplasmic faces of the NPC. The exception was the nuclear basket analog TbNup110, which confirmed the ability of our approach to reveal asymmetric localizations. Moreover, clear homologs of Nups and accessory transport factors with asymmetric nucleocytoplasmic distributions on the NPC were absent from our affinity captures and from exhaustive informatics screens of the trypanosome genome. Taken together, our data therefore indicates that, with the exception of the nuclear basket, the trypanosome NPC lacks a clear nucleoplasmic- or cytoplasmic-biased localization of Nups, in contrast to opisthokonts (Fig 7A). One source of Nup asymmetry in opisthokonts is from ScNup145/HsNup98-96, which can self-cleave to release an N-terminal fragment (ScNup145N) that localizes preferentially to the nuclear side of the NPC. Intriguingly, ScNup145N facilitates the connection between inner and outer ring complexes via discrete binding motifs for inner ring, central channel, and cytoplasmic Nups [112]. In contrast, TbNup158, the ortholog of this protein in trypanosomes, lacks the catalytic residues required for autoproteolytic cleavage to generate FG-Nup (ScNup145N/HsNup98) and α-solenoid Nup (ScNup145C/HsNup96) fragments [27,113–115]. Thus, FG-Nup symmetry is maintained by ensuring that TbNup158 is incorporated into the TbNPC as a single FG/α-solenoid protein in the symmetrically disposed outer ring complex. In addition, TbNup76, orthologous to ScNup82/HsNup88, is located on both the cytoplasmic and nucleoplasmic faces of the trypanosome NPC, but is part of an exclusively cytoplasmic NPC subcomplex in opisthokonts (Fig 7A). Furthermore, the FG-Nup ScNsp1/HsNup62 is also present in two distinct NPC subcomplexes; the inner ring ScNic96/HsNup93 complex and the cytoplasmic ScNup82/HsNup88 complex [87,116], thus representing another form of Nup asymmetry in opisthokont NPCs. In contrast, none of the potential trypanosome orthologs of ScNsp1/HsNup62 appears to associate with another TbNPC subcomplex, further highlighting the distinct symmetry exhibited by the TbNPC.

The symmetric arrangement in trypanosomes is also consistent with the hypothesis that the basic mechanism of nucleocytoplasmic transport does not require inherent NPC asymmetry [6,13]. However, it is significant that while trypanosomes share a diverse array of FG-Nup “flavors” with opistokhonts, in trypanosomes, this does not correlate strongly with their nucleocytoplasmic arrangement (S4 Table).

### A Putative Role for the GTPase Ran in mRNA Export

The main mRNA export factor Mex67 and its partner Mtr2 are conserved in trypanosomes, consistent with previous observations that karyopherin transport factors are also well conserved [117]. Given this evolutionary conservation of transport factors, there is, a priori, no reason to suspect major differences in transport mechanisms in trypanosomes. However, the cytoplasmically disposed, ATP-powered mRNA export platform formed by the ScNup82/HsNup88 complex, specifically ScNup159/HsNup214 plus the export factors Gle1 and the ATP-dependent helicase Dbp5 in opisthokonts [22,118,119], appears almost entirely lacking in trypanosomes. Therefore, in the absence of this cytoplasmic ATPase assembly, how is mRNA export both powered and provided with directionality in trypanosomes? A possible mechanism is suggested by affinity captures of Mex67, which recovered stoichiometric amounts of the GTPase Ran, RanBP1, and a putative GTPase activating protein, even though neither yeast nor vertebrate Mex67 or Mtr2 bind Ran [40,106]. Previous work has suggested that trypanosome mRNA export may be mechanistically distinct from that in opisthokonts and plants, with a shared platform for transport of rRNA and mRNA [30,120]. Here, our data strongly suggest that mRNA export in trypanosomes is dependent for both directionality and energy on the GTPase Ran, similar to karyopherin-mediated transport (Fig 7B). In opisthokonts, Ran, RanBP1, and a RanGAP are normally involved in an exquisite interplay that promotes hydrolysis of RanGTP to RanGDP, facilitating cargo release into the cytoplasm [121–123], and perhaps an analogous mechanism is involved in trypanosome mRNA export. Trypanosomes have rather unusual mechanisms for controlling gene expression, possibly a reflection of early divergence that places them close to the eukaryotic root [124,125]. Trypanosome protein-coding genes lack introns and are organized into directional polycistronic transcription units (PTUs) comprised of functionally unrelated genes [126,127]. Each gene lacks an individual promoter, with transcription start and stop sites only present for the entire PTU [128]. PTUs are transcribed by RNA polymerase II into long polycistronic transcripts, and the processing of single mRNAs is achieved by *trans*-splicing and subsequent polyadenylation, with regulation of gene expression therefore relying mainly on mRNA turnover and translation rates [129,130]. This exclusive *trans*-splicing of protein-coding mRNAs in trypanosomes may remove much of the complexity of mRNA processing, relaxing requirements for extensive chaperoning or quality control during nuclear export, and so accounting for the differences we find between the opisthokont and the kinetoplastid mRNA export machineries. It is appealing to propose that trypanosome may exemplify (or may have reinvented) an ancestral configuration for nucleocytoplasmic transport, whereby all transport factors operated in a Ran-dependent manner, but this remains tentative at this time.

### Implications for the Evolutionary Origins of the NPC

The origin of an NE that defines the nucleoplasm necessitated development of an exchange mechanism with the cytoplasm. Hence, the NPC must, at least in part, embody this major transition in cellular architecture. Despite 1.5 billion years separation, animals, fungi, plants, and trypanosomes all utilize the NPC for nucleocytoplasmic transport, plus mRNA processing and maintenance of the chromatin environment. While the NPC demonstrates significant subunit conservation across eukaryotes, the manner in which the NPC connects with the lamina and mRNA transport is likely highly divergent between these lineages [35,42].

The trypanosome NPC architecture supports our earlier model of NPC evolution, which proposed that the ancestral NPC was an ungated pore, with protocoatomer type subunits stabilizing fenestrations in the protoeukaryotic NE [38]. Conservation of the core scaffold, and the presence of the same folds throughout the scaffold, supports a basic tenet of this model, i.e., that the elaborate architecture of the NPC arose through repeated duplication events from a simple progenitor coating complex. Even the eight-fold symmetry, conserved in trypanosomes [131], suggests a model for a stepwise monomer to dimer to tetramer to octamer transition during evolution. Of significance is that membrane anchoring of protocoatomer systems is promiscuous [11], consistent with divergent NPC membrane tethering described here. Selective gating by FG-Nups was proposed as a more recent acquisition, facilitating more selectivity in import and export [27]. Nevertheless, the high degree of conservation found in the inner ring complex, which contains representatives of all the major elements of the transport machinery (coatomer, karyopherin, FG Nup, membrane association), suggests an intermediate but simpler architecture for a transitional pre-LECA NPC. We propose that, subsequently, a more elaborate architecture evolved, leading to differentiated inner and outer rings and peripheral structures, and providing specific and different functionalities at the nuclear versus cytoplasmic sites. This allowed the development, in particular, of elaborations in mRNP processing and assembly at the NPC's nucleoplasmic face and ATP-dependent export and unloading on the cytoplasmic face. This may also have driven remodeling of FG-Nup positioning, with the trypanosome symmetric arrangement perhaps reflecting that in the LECA NPC, and being consistent with the trypanosomatid lineage as one of the earliest to differentiate following the eukaryogenesis event.

## Methods

### Cell Culture

*T*. *brucei* procyclic Lister 427 strain cells were cultured in SDM-79, supplemented with 10% fetal bovine serum as previously described [27,132]. Expression of plasmid constructs was maintained using Hygromycin B at 30 μg/ml.

### In Situ Genomic Tagging

All proteins tagged in this study used the pMOTag4G tagging vectors [133] as previously described [27].

### Fluorescence Microscopy

GFP-tagged cell lines were harvested and fixed for 10 mins in a final concentration of 2% paraformaldehyde. Fixed cells were then washed in 1xPBS and visualized as previously described [27].

### Affinity Isolation

Trypanosomes were grown to a density of between 2.5 x 10^7^ cells per ml. Parasites were harvested by centrifugation, washed in 1xPBS with protease inhibitors and 10mM dithiothreitol, and flash frozen in liquid nitrogen to preserve protein:protein interactions as close as they were at time of freezing as possible. Cells were cryomilled into a fine grindate in a planetary ball mill (Retsch). For a very detailed protocol, refer to Obado et al., 2015 (in press), Methods in Molecular Biology, or the National Center for Dynamic Interactome Research website (www.NCDIR.org/protocols). Cryomilled cellular materials were resuspended in various extraction buffers (S1 Table) containing a protease inhibitor cocktail without EDTA (Roche), sonicated on ice with a microtip sonicator (Misonix Ultrasonic Processor XL) at Setting 4 (~20W output) for 2 x 1 second to break apart aggregates that may be invisible to the eye, and clarified by centrifugation (20,000 x *g*) for 10 min at 4°C (Obado et al., 2015 (in press), Methods in Molecular Biology, or www.NCDIR.org/protocols) [41]. Clarified lysates were incubated with magnetic beads conjugated with polyclonal anti-GFP llama antibodies on a rotator for 1 hr at 4°C. The magnetic beads were harvested by magnetization (Dynal) and washed three times with extraction buffer prior to elution with 2% SDS/40 mM Tris pH 8.0. The eluate was reduced in 50 mM DTT and alkylated with 100 mM iodoacetamide prior to downstream analysis (SDS-PAGE followed by protein identification using MS—electrospray ionization (ESI) or MALDI-TOF). Eluates were fractionated on precast Novex 4–12% Bis Tris gels (Life Technology), stained using colloidal Coomassie (GelCode Blue—Thermo) and analyzed by MS [27].

### Mass Spectrometry

Briefly, protein bands were excised from acrylamide gels and destained using 50% acetonitrile, 40% water, and 10% ammonium bicarbonate (v/v/w). Gel pieces were dried and resuspended in trypsin digestion buffer; 50 mM ammonium bicarbonate, pH 7.5, 10% acetonitrile, and 0.1–2 ug sequence-grade trypsin, depending on protein band intensity. Digestion was carried out at 37°C for 6 h prior to peptide extraction using C18 beads (POROS) in 2% TFA (trifluoroacetic acid) and 5% formamide. Extracted peptides were washed in 0.1% acetic acid (ESI) or 0.1% TFA (MALDI) and analyzed on a LTQ Velos (ESI) (Thermo) or pROTOF (MALDI-TOF) (PerkinElmer).

### Secondary Structure Prediction

Newly identified TbNups were analyzed for several secondary structure elements, including β-sheets and α-helices using PSIPRED [134] and Phyre2 [57], natively unfolded regions using Disopred [135], *trans*-membrane helices using Phobius [136], and coiled-coil regions using COILS [137].

### Immunogold Labeling

Trypanosomes were cryoprotected with 20% bovine serum albumin (BSA) and applied to a high pressure freezing procedure (EMPACT2, Leica Microsystem System, Wetzlar, Germany). Cells were transferred to a freeze substitution device (EM AFS2, Leica Microsystem System, Wetzlar, Germany), incubated with 0.2% Uranyl acetate in 95% acetone at -90 C°, and embed in Lowicryl HM20 at -35 C°. Ultrathin sections were cut and post-embedding immunostaining was applied. Briefly, sections were blocked with 2% BSA plus 0.1% saponin in Tris buffered saline (TBS; 20 mM Tris-Cl, pH 7.5, 150 mM NaCl) for 30 min. Sections were then incubated in fresh blocking solution containing polyclonal rabbit anti-GFP antibodies (1:150) overnight at 4°C, and washed with TBS the next day. The EM sections were then incubated overnight with secondary goat anti-rabbit antibodies conjugated with 12 nm colloidal gold (1:20) in 0.2% BSA plus 0.1% saponin in TBS and then washed in TBS buffer. An additional wash step using 1 x PBS was performed prior to fixation for 5 min with 2.5% glutaraldehyde. Post fixed grids were washed with water and uranyl acetate (1%), and lead citrate (1%) was applied. The sections were examined in the electron microscope (100CX JEOL, Tokyo, Japan) with the digital imaging system (XR41-C, AMT Imaging, Woburn, Massachusetts). Control experiments were done by following the same procedure, except for the omission of primary antibody and applying just the blocking solution instead.

### Immunoelectron Microscopy Montages

We selected NPCs sectioned perpendicular to the NE plane with a clearly visible nuclear envelope double membrane. We selected a radius of 300 nm around the estimated center of each NPC as an excision limit and then created an aligned superimposed montage using the resulting excised NPC images [6,36]. See S2 Fig. For the radial position of each Nup (R), we used the method described in [58] and the peak finding algorithm of Alber et al., 2007 [3,4]. For the axial position of each Nup (Z), we essentially used the method described in [3,4]; for both, errors were estimated from the 95% level of the peak finding algorithm.

### Sodium Carbonate Extraction and Western Blot

Powder from cryomilled trypanosomes was resuspended in 0.1 M Na-Carbonate buffer, pH 11 to a ratio of 1:9 (powder:buffer) and then processed as previously described [6].

### Structural Modeling

3D structures were modeled using the program I-TASSER [105,138], which combines fold recognition, where the template is threaded onto similar structures retrieved from the pdb, full length reconstruction of the template involving ab initio modeling of unaligned regions and rigorous high-resolution refinement to generate a final protein model. For our studies, no threading templates from the pdb were specified; instead, we chose to employ the default search criteria on the I-TASSER server for template threading. All models were viewed and figures generated using PyMOL (The PyMOL Molecular Graphics System, Version 1.7.4 Schrödinger, LLC.).

## Supporting Information

S1 Fig Raw, one-dimensional protein electrophoresis of affinity isolated TbNup complexes and determined protein identities. Besides nucleoporins, we identified many known contaminants (Llama IgG heavy, light, and variant chains), highly abundant proteins such as tubulin and heat shock proteins, and putative NE and nuclear basket associated proteins were identified by mass spectrometry. Proteins represented by Gene IDs Tb927.7.4760, Tb927.9.6460, Tb927.6.890, Tb927.8.3950, and Tb927.9.1410 were tagged and affinity isolated but did not exclusively co-isolate known TbNups. These are under investigation. Tb927.4.2850 (putative RNA binding protein) and Tb927.11.550 (orthologous to yeast SCD6 protein) were not investigated. Tb927.7.6670 and Tb927.9.11150 were refractory to GFP tagging.
(TIF)

S2 Fig Example of building up immuno-EM montages. Only NPCs sectioned perpendicular to the NE plane with a clearly visible double membrane, and where the position of the NPC and NE are clear, are selected. We then selected a radius of 300 nm around the estimated center of each NPC as an excision limit and created a superimposed montage using the resulting excised NPC images and the position of the NE/NPC electron density as reference [6,36,58]
(TIF)

S3 Fig Immuno-gold labeled iEM montages and *y*-axis histogram plots. GFP-tagged Nups were immuno-gold labeled using polyclonal anti-GFP rabbit antibodies (Methods). We picked NPCs sectioned perpendicular to the NE plane and selected a radius of 300 nm around the estimated center of each NPC and excised each image (S1 Fig). We then aligned and created a superimposed montage of several excised NPC images [6,36]. Y positions of each gold particle were measured relative to the NPC midplane and plotted as a histogram with each segment representing a distance of 10 nm (See S1 File).
(TIF)

S4 Fig *Trans*-membrane domain prediction of trypanosome and leishmania Nup65 homologs. The software Phobius (http://phobius.sbc.su.se/) [136] was used to identify putative *trans-*membrane domains in kinetoplastid homologs of TbNup65.
(TIF)

S5 Fig Tb927.7.4760 localizes to both the nuclear rim and cytoplasmic puncta reminiscent of the Golgi. Tb927.7.4760 was tagged in situ with GFP [133]. Panels show trypanosomes in phase contrast and with GFP visualized directly (Methods).
(TIF)

S6 Fig Phylogenetic tree comparing outer ring complex beta propeller proteins. All known beta propeller proteins from yeast and mammalian NPCs were used to search the *T*. *brucei* genome. The top three hits in each case were retained. All sequences were then combined, and redundancies were removed. The trypanosome, yeast, and mammalian sequences were then aligned using Clustal and the alignment masked to exclude regions of high divergence, typically extensive indels. The alignment was then used to build a phylogenetic tree using both MrBayes and PhyML. The MrBayes topology is shown. Taxa are color coded and the statistical support for each node shown as indicated in the key.
(TIF)

S7 Fig Nup149 is a protein comprising three repeat domains. (A) TbNup149 is comprised of three repetitive domains as shown. This repetitive feature is conserved in other kinetoplastids (not shown). Putative zinc finger domains are underlined and highlighted in black. FG domains are marked in red and the beginning and end of each repeat marked in green. (B) An alignment comparing the protein sequence of each repeated domain. (C) A comparison between the nucleotide sequence of each repeat. The nonrepeated segments of TbNup149 are not compared.
(DOCX)

S8 Fig The ortholog of the ATP-dependent DEAD box helicase Dbp5 appears to be absent from the trypanosome genome. (A) A panel of 50-proteomes was scanned using PSI-BLAST with the pfam DEAD domain (methodology same as in O’Reilly et al., 2011). All hits with e-value LT 0.0001 were collected and a Neighbor Joining (NJ) tree was constructed using the domain sequences only. This tree was then annotated with protein sequence length and hmmscan predictions (e-value LT 0.1) for full-length sequence. The DBP5 cluster (59 members) is at the bottom end of the tree with no evident trypanosome sequences present. (B) A reciprocal best hit BLAST was run across the panel of all 59 DBP5 candidates to test if any of them could pull out trypanosome sequences. None of the 59 candidates gave a rbhBLAST hit in the trypanosomes. Forward BLAST results from the above rbhBLAST scan identified top hits from trypanosomes that were mostly from a specific trypanosome clade (the clade containing Tb11.12.0011) that is not part of an all-eukaryote clade being on the outside of the IF4A clade. An rbhBLAST scan with these sequences determined that they correspond to the DEAD subfamily “FAL1,” suggesting that there are no DBP5 orthologs in trypanosomes. The IF4A, FAL1, and DBP5 are in this order about three-quarters of the way down the tree.
(ZIP)

S1 File X and Y gold positions and Y histograms of selected TbNups for immunoelectron microscopy. Individual files for each TbNup that show X and Y gold positions for each TbNup. The histograms were plotted using *y*-axis gold positions relative to the NPC midplane (see S3 Fig).
(XLSX)

S1 Table Interactome of the TbNPC with corresponding extraction buffer conditions. A table showing each TbNup (blue) and the identified interacting partners. Affinity isolation buffers are also indicated for each TbNup, including the RNA export factor TbMex67. The peach color on the label represents outer ring Nups. Purple = inner ring α-solenoids and β-α Nups, blue and pink represent the linker Nups, green = FG-Nups, yellow = nuclear basket Nups, and white = TbNup48/ALADIN, which was not characterized in this study due to our inability to find co-isolating Nups despite testing several affinity isolation conditions.
(XLSX)

S2 Table Statistical analysis of relative positions of select TbNups biy immuno-gold labeling. X and Y positions of gold particles from iEM montages in S3 Fig were measured, from which the Z and R (cylindrical Rotational axis of the NPC) axes were calculated. Z average values are positive or negative to represent localizations above and below the midplane of the NPC. The relative location of each Nup was plotted based on the R and Z values whose axes errors are plotted based the 95% level of a peak finding algorithm [6]. Abbreviations: ave (average), Err (error), N(R) (number of gold particles used to calculate the R-axis), N(Z) (number of gold particles used to calculate the Z-axis).
(XLSX)

S3 Table A comparison between the relative molecular weights of Nups across well characterized taxa. Inner ring Nups are very similar in size in opisthokonts (represented here by yeast and humans), trypanosomes, and green plants (Arabidopsis). This may reflect constraints in building a cylindrical channel through the NE that is circa 50 nm in length and delimits a central channel 40 nm wide. Indeed, the entire scaffold (outer and inner ring) appears well conserved by size. Major differences between the trypanosomes and other taxa lie in the nuclear basket, which is half the size, and the absence of Poms. Orthologs of FG-Nups between trypanosomes, opisthokonts, and plants are not easily defined and are not compared.
(XLSX)

S4 Table The major type of flavors found in trypanosome FG-Nups. There appear to be three major FG-Nup flavors in trypanosomes. Interestingly, the inner and outer ring FG-Nups all share the same GFG flavor. Likewise, the two Nup76 FG-Nups have SVFG or PAFG flavors (predominantly PAFG for Nup140). The multi complex FG-Nups also have a shared FSFG flavor.
(DOCX)

## Abbreviations

ALPS: amphipathic lipid-packing sensor
DAPI: 6-diamino-2-phenylindoledihidrochloride
FG: phenylalanine-glycine
GAP: GTPase Activating Protein
GFP: green fluorescent protein
iEM: immunoelectron microscopy
IP_6_: inositol hexakisphosphate
mRNP: messenger ribonucleoprotein
MS: mass spectrometry
NE: nuclear envelope
NPC: nuclear pore complex
Nups: nucleoporins
Pel: pellet
Poms: pore membrane proteins
PTU: polycistronic transcription units
RRM: RNA recognition motif
Sup: supernatant
TbNPC: *T*. *brucei* NPC
TbNups: *T*. *brucei* Nups
TM: *trans*-membrane domain
