# Supplementary figures and images for: Interactome Mapping Reveals the Evolutionary History of the Nuclear Pore Complex
Source: PLoS Biol. 2016 Feb 18;14(2):e1002365. doi: 10.1371/journal.pbio.1002365 (PMC4758718; doi:10.1371/journal.pbio.1002365)

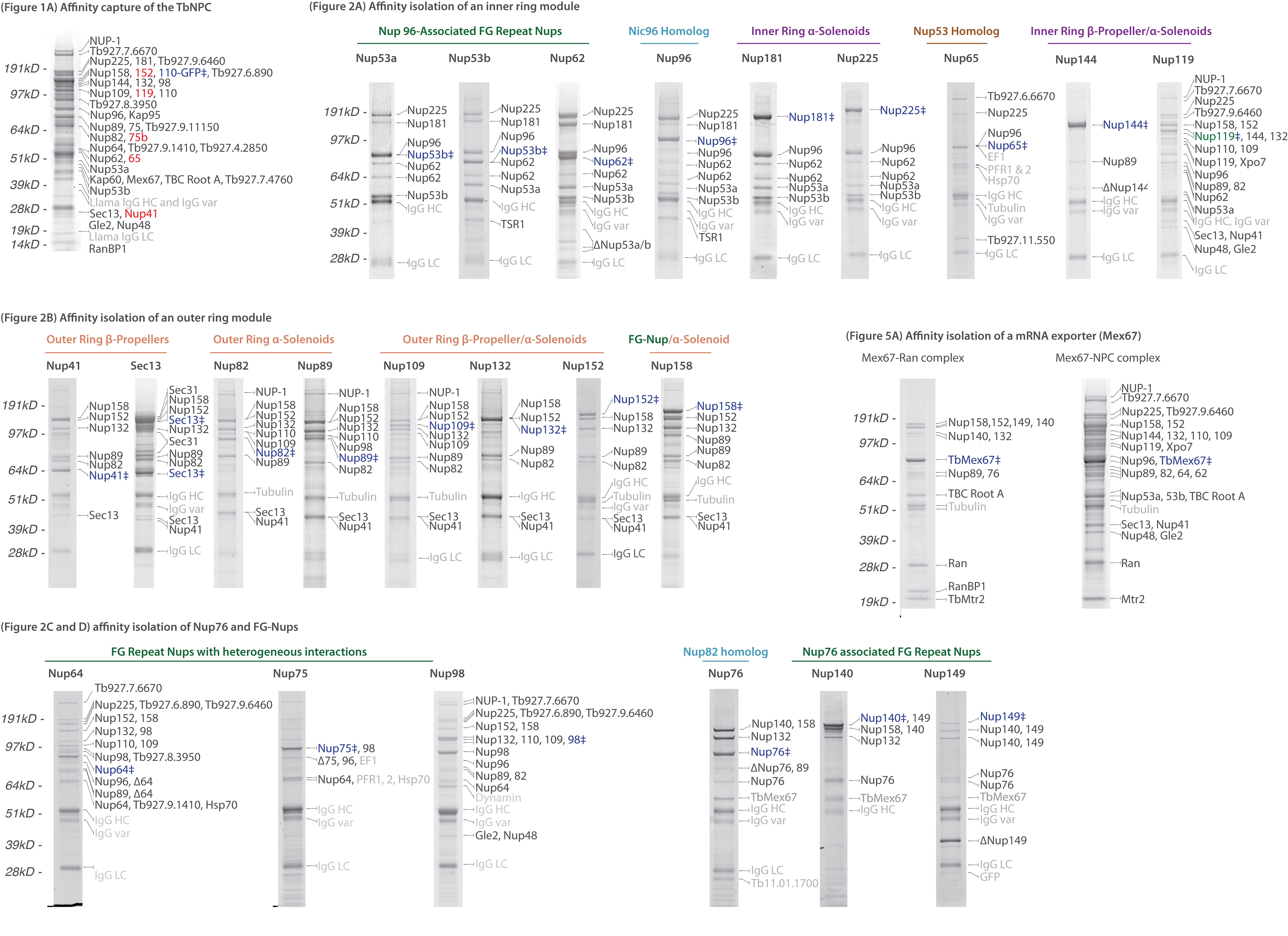

Supplement: S1 Fig — Besides nucleoporins, we identified many known contaminants (Llama IgG heavy, light, and variant chains), highly abundant proteins such as tubulin and heat shock proteins, and putative NE and nuclear basket associated proteins were identified by mass spectrometry. Proteins represented by Gene IDs Tb927.7.4760, Tb927.9.6460, Tb927.6.890, Tb927.8.3950, and Tb927.9.1410 were tagged and affinity isolated but did not exclusively co-isolate known TbNups. These are under investigation. Tb927.4.2850 (putative RNA binding protein) and Tb927.11.550 (orthologous to yeast SCD6 protein) were not investigated. Tb927.7.6670 and Tb927.9.11150 were refractory to GFP tagging. (TIF) [file pbio.1002365.s001.tif]

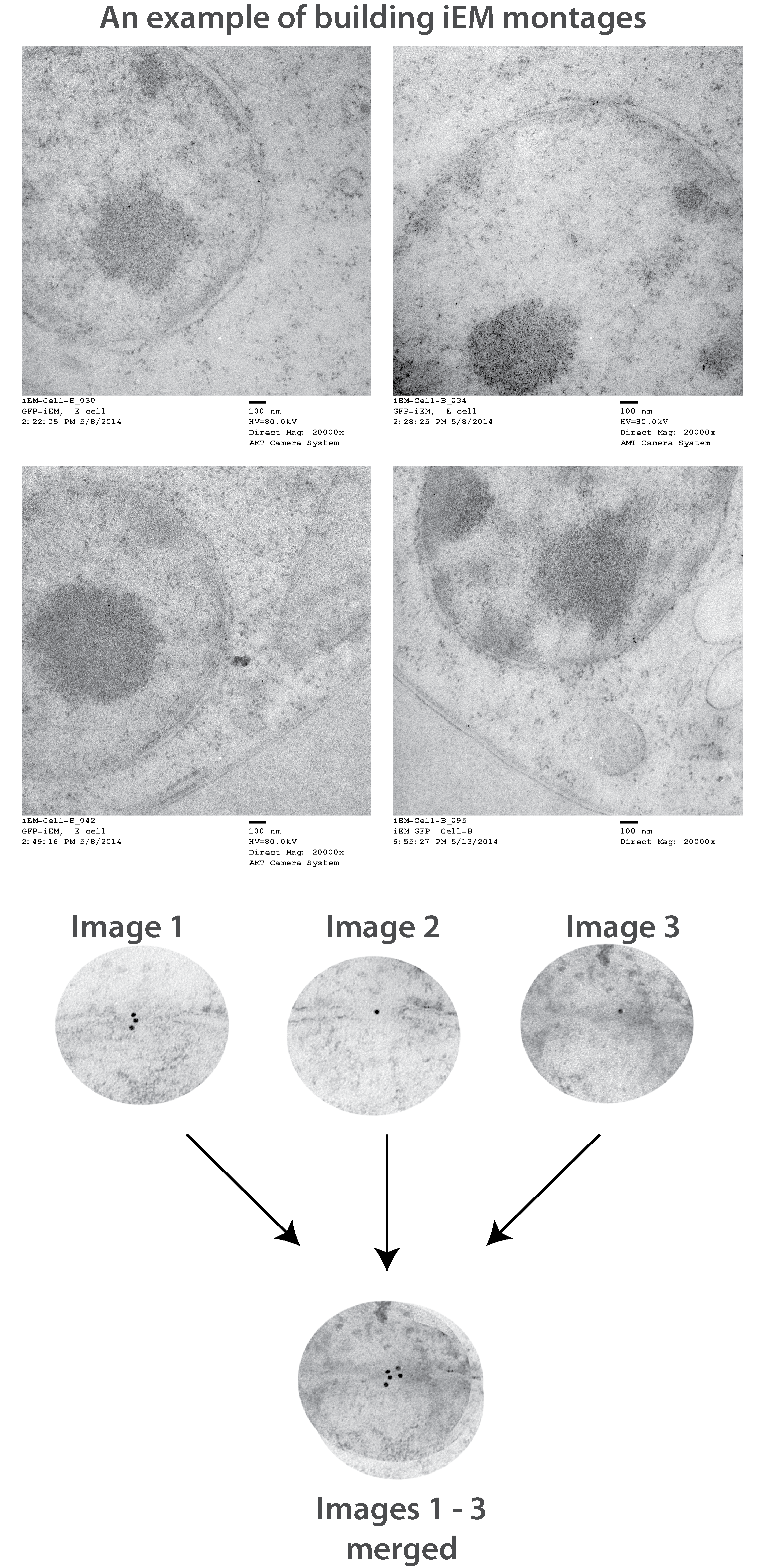

Supplement: S2 Fig — Only NPCs sectioned perpendicular to the NE plane with a clearly visible double membrane, and where the position of the NPC and NE are clear, are selected. We then selected a radius of 300 nm around the estimated center of each NPC as an excision limit and created a superimposed montage using the resulting excised NPC images and the position of the NE/NPC electron density as reference [6,36,58] (TIF) [file pbio.1002365.s002.tif]

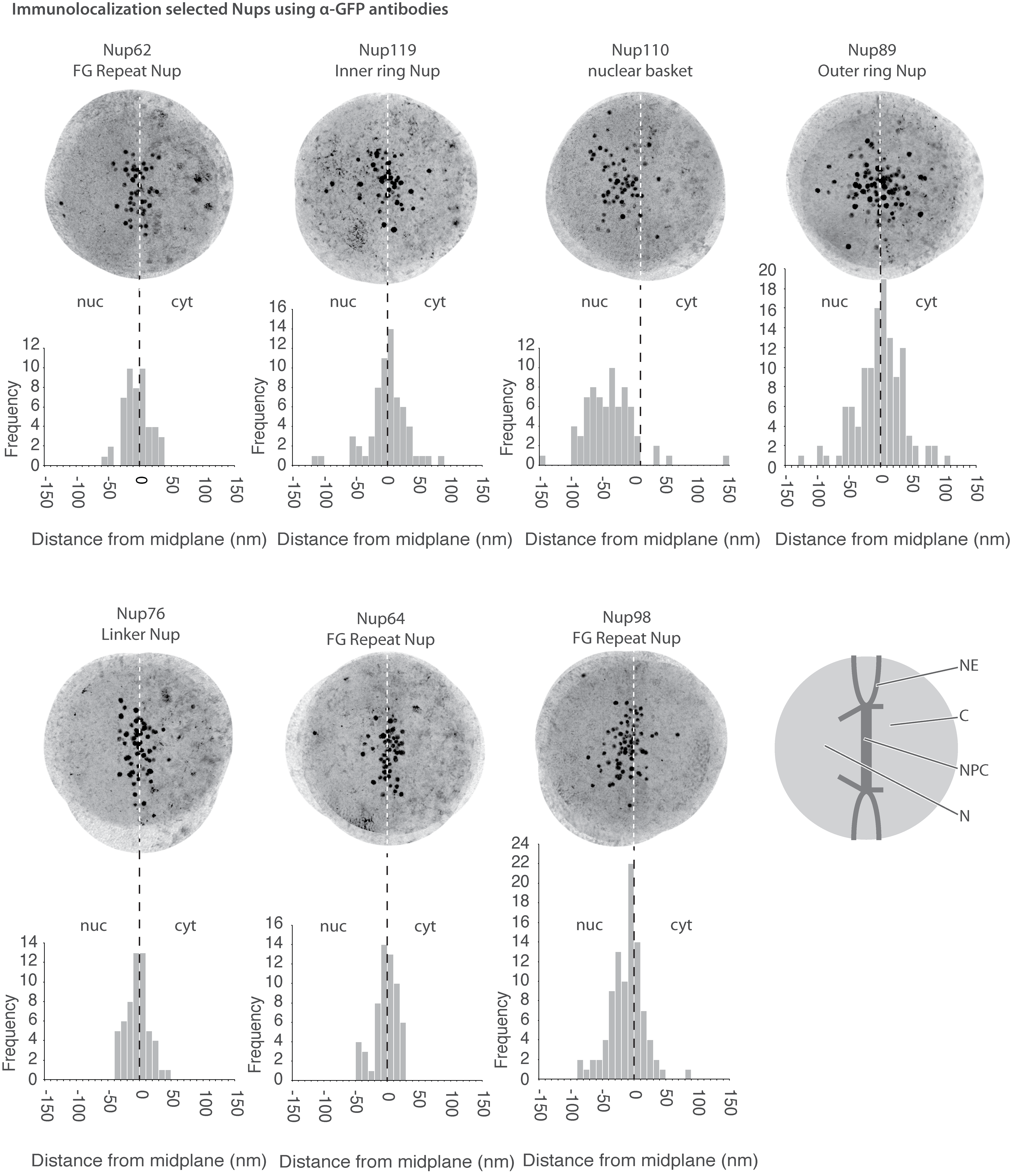

Supplement: S3 Fig — GFP-tagged Nups were immuno-gold labeled using polyclonal anti-GFP rabbit antibodies (Methods). We picked NPCs sectioned perpendicular to the NE plane and selected a radius of 300 nm around the estimated center of each NPC and excised each image (S1 Fig). We then aligned and created a superimposed montage of several excised NPC images [6,36]. Y positions of each gold particle were measured relative to the NPC midplane and plotted as a histogram with each segment representing a distance of 10 nm (See S1 File). (TIF) [file pbio.1002365.s003.tif]

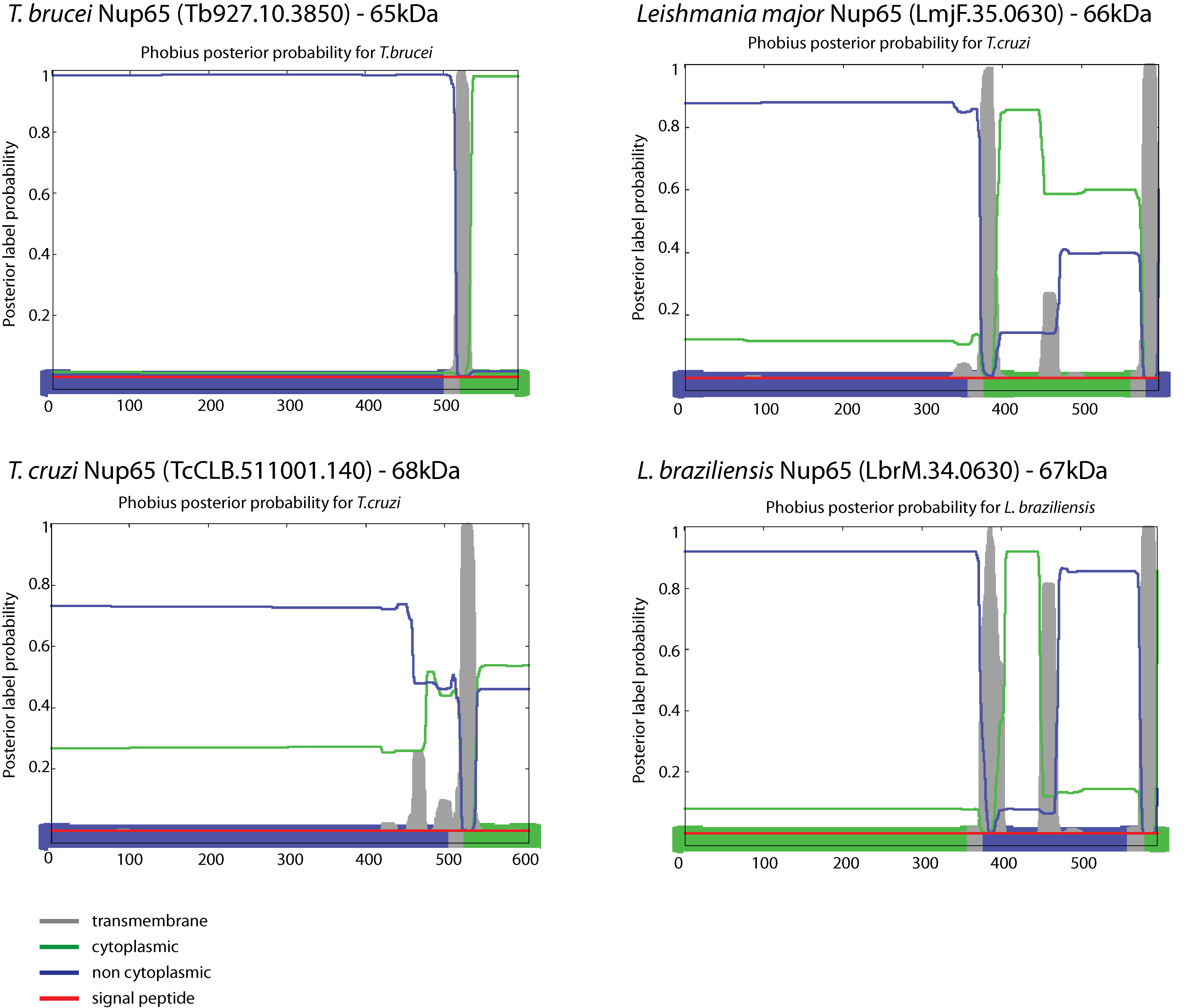

Supplement: S4 Fig — The software Phobius (http://phobius.sbc.su.se/) [136] was used to identify putative trans-membrane domains in kinetoplastid homologs of TbNup65. (TIF) [file pbio.1002365.s004.tif]

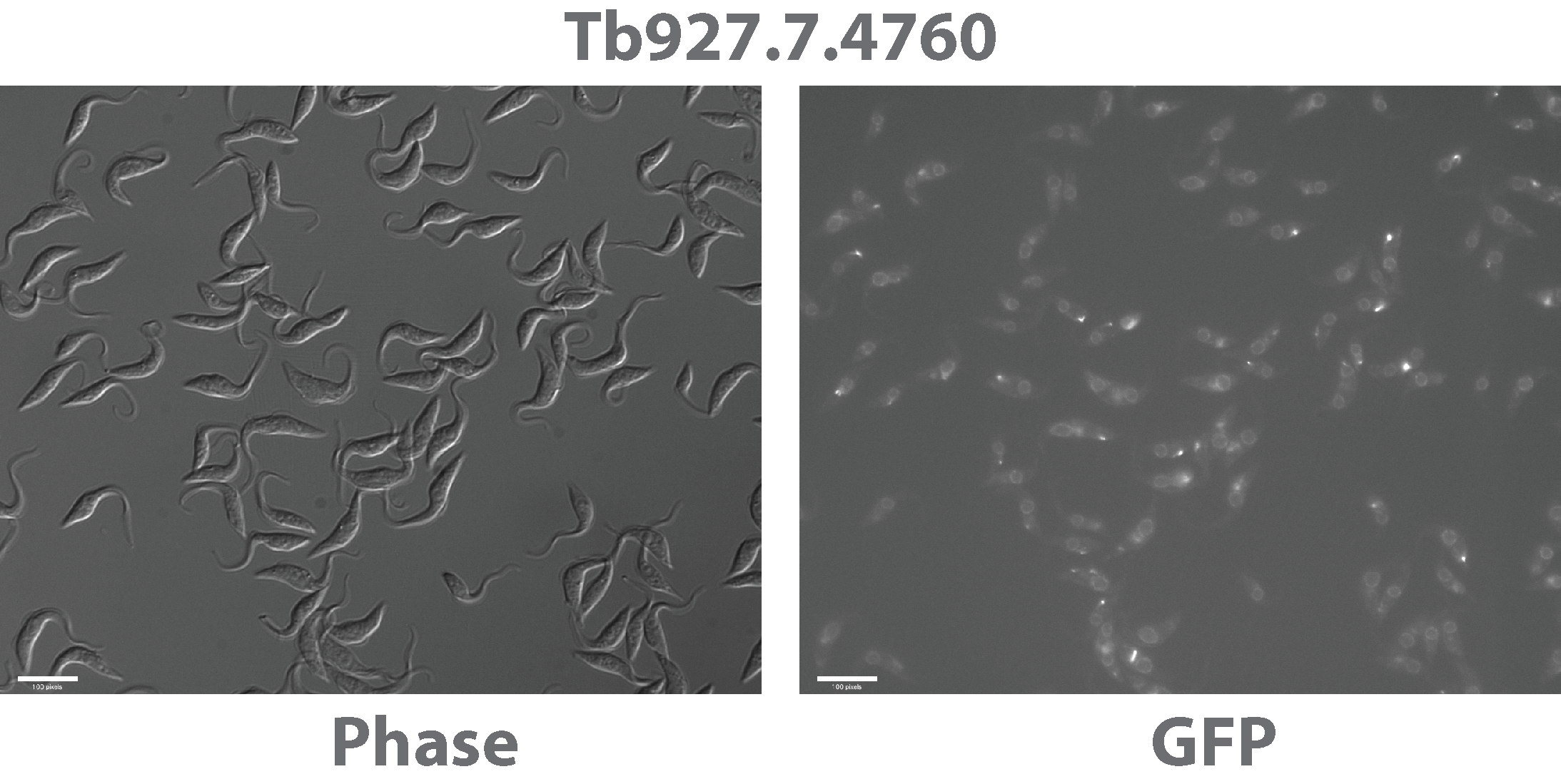

Supplement: S5 Fig — Tb927.7.4760 was tagged in situ with GFP [133]. Panels show trypanosomes in phase contrast and with GFP visualized directly (Methods). (TIF) [file pbio.1002365.s005.tif]

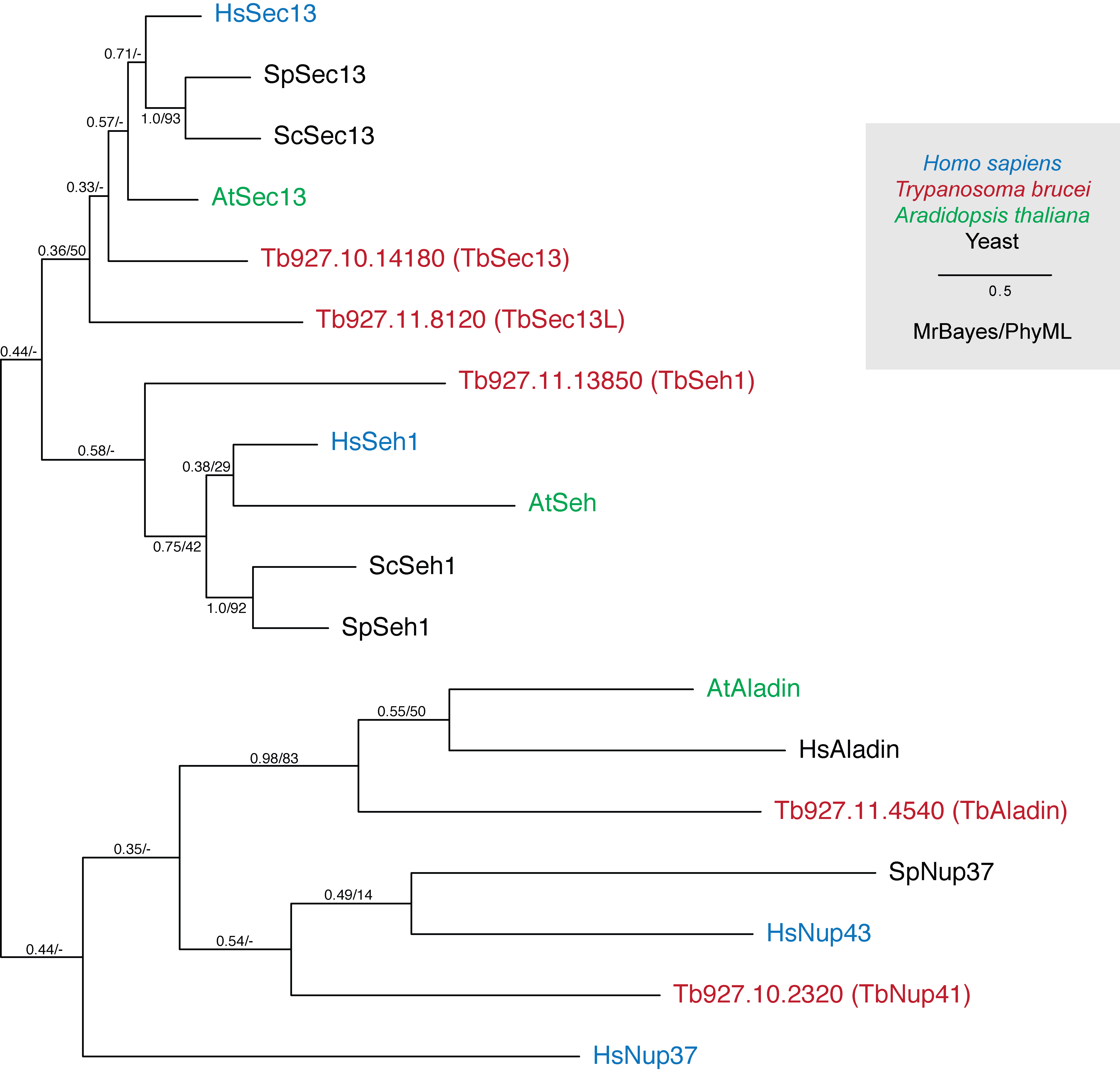

Supplement: S6 Fig — All known beta propeller proteins from yeast and mammalian NPCs were used to search the T. brucei genome. The top three hits in each case were retained. All sequences were then combined, and redundancies were removed. The trypanosome, yeast, and mammalian sequences were then aligned using Clustal and the alignment masked to exclude regions of high divergence, typically extensive indels. The alignment was then used to build a phylogenetic tree using both MrBayes and PhyML. The MrBayes topology is shown. Taxa are color coded and the statistical support for each node shown as indicated in the key. (TIF) [file pbio.1002365.s006.tif]
