# Supplementary material for: Interactome Mapping Reveals the Evolutionary History of the Nuclear Pore Complex
Source: PLoS Biol. 2016 Feb 18;14(2):e1002365. doi: 10.1371/journal.pbio.1002365 (PMC4758718; doi:10.1371/journal.pbio.1002365)
Supplement: S7 Fig — (A) TbNup149 is comprised of three repetitive domains as shown. This repetitive feature is conserved in other kinetoplastids (not shown). Putative zinc finger domains are underlined and highlighted in black. FG domains are marked in red and the beginning and end of each repeat marked in green. (B) An alignment comparing the protein sequence of each repeated domain. (C) A comparison between the nucleotide sequence of each repeat. The nonrepeated segments of TbNup149 are not compared. (DOCX) [file pbio.1002365.s007.docx]

**(A) Full length TbNup149 with repeated segments, FG regions and putative zinc fingers highlighted for clarity.**

**MSEIAHSAVGDVPIDSFKADSHNGGKDAWVCTRCGKVKDLRGDHLRGKMEVRSDCWPCGKKATFKLQSRLSDDGSNNNVPAASTVDDTHQPKGVGNATIVGGFRAPVVPSTGDEHQFLFPAPVLQCSTPSLSADCSSVWKCSICGKVKPLSGDHLRGKTEVRSDCWPCGCKRTFVLSPVDGEYGCSALPKEDASKTISKASENVKEQATTGAQIPVQPAAIPFKSVFDTQSSGDAKPPAATPFKSVFDTQSSGDAKPPAATPFKSVFGTQSSGDAKPPAATPFKSVFGTQSSGDAKPPAATPFKSVFDTQSSGDAKPPAATPFKSVFDTQSSGDAKPPAATPFKSVFGTQSSGDAKPPAATPFKSVFDTQSSGDAKPPAATPFKSVFGTQSSGDAKPPAATPFKSVFDTQSSGDAKPPAATPFKSDSINGFFIKQTRAGI**

**MSEIAHSAVGDVPIDSFKADSHNGGKDAWVCTRCGKVKDLRGDHLRGKMEVRSDCWPCGKKATFKLQSRLSDDGSNNNVPAASTVDDTHQPKGVGNATIVGGFRAPVVPSTGDEHQFLFPAPVLQCSTPSLSADCSSVWKCSTCGKVKPLSGDHLRGKTEVRSDCWPCGCKRTFVLSPVDGEYGCSALPKEDASKTISKASENVKEQATTGAQIPVQPAAIPFKSVFDTQSSGDAKPPAATPFKSVFGTQSSGDAKPPAATPFKSVFGTQSSGDAKPPAATPFKSVFGTQSSGDAKPPAATPFKSDSINGFFIKQTRAGI**

**MSEIAHSAVGDVPIDSFKADSHNGGKDAWVCTRCGKVKDLRGDHLRGKMEVRSDCWPCGKKATFKLQSRLSDDGSNNNVPAASTVDDTHQPKGVGNATIVGGFRAPVVPSTGDEHQFLFPAPVLQCSTPSLSADCSSVWKCSTCGKVKPLSGDHLRGKTEVRSDCWPCGCKRTFVLSPVDGEYGCSALPKEDASKTISKASENVKEQATTGAQIPVQPAATPFKSVFDTQSSGDAKPPAATPFKSVFGTQSSGDAKPPAATPFKSVFGTQSSGDAKPPAATPFKSVFDTQSSGDAKPPAATPFKSVFGTQSSGDAKPPAATPFKSVFDTQSSGDAKPPAATPFKSVFGTQSSGDAKPPAATPFKSVFGTQSSGDAKPPAATPFKSVFGTQSSGDAKPPAATPFKSVFDTQSSGDAKPPAATPFKSVFDTQSSGDAKPPAATPFKSVFGTQSSGDAKPPAATPFKSVFGTQSSGDAKPPAATPFKSVFGTQSSGDAKPPAATPFKSVFGTQSSGDAKPPAATPFKSVFGTQSSGDANCRGGVVSICLDGESSVTNSASSSCRSTVQANILHDSVGGGVVCVADDLSGYQSNSLDEALNVTGLSPLFSAFERMMNDAKEKVLSALKEELNQMVSCVGGRDCCKCGRAIQSGREVKENYRGRNKSGDKIMWAHLENAFTKPY**

**(B) Alignment of the three repeated segments of TbNup149**

**Rpt 1 MSEIAHSAVGDVPIDSFKADSHNGGKDAWVCTRCGKVKDLRGDHLRGKMEVRSDCWPCGK 60**

**Rpt 2 MSEIAHSAVGDVPIDSFKADSHNGGKDAWVCTRCGKVKDLRGDHLRGKMEVRSDCWPCGK 60**

**Rpt 3 MSEIAHSAVGDVPIDSFKADSHNGGKDAWVCTRCGKVKDLRGDHLRGKMEVRSDCWPCGK 60**

****************************************************************

**Rpt 1 KATFKLQSRLSDDGSNNNVPAASTVDDTHQPKGVGNATIVGGFRAPVVPSTGDEHQFLFP 120**

**Rpt 2 KATFKLQSRLSDDGSNNNVPAASTVDDTHQPKGVGNATIVGGFRAPVVPSTGDEHQFLFP 120**

**Rpt 3 KATFKLQSRLSDDGSNNNVPAASTVDDTHQPKGVGNATIVGGFRAPVVPSTGDEHQFLFP 120**

****************************************************************

**Rpt 1 APVLQCSTPSLSADCSSVWKCSICGKVKPLSGDHLRGKTEVRSDCWPCGCKRTFVLSPVD 180**

**Rpt 2 APVLQCSTPSLSADCSSVWKCSTCGKVKPLSGDHLRGKTEVRSDCWPCGCKRTFVLSPVD 180**

**Rpt 3 APVLQCSTPSLSADCSSVWKCSTCGKVKPLSGDHLRGKTEVRSDCWPCGCKRTFVLSPVD 180**

************************ ***************************************

**Rpt 1 GEYGCSALPKEDASKTISKASENVKEQATTGAQIPVQPAAIPFKSVFDTQSSGDAKPPAA 240**

**Rpt 2 GEYGCSALPKEDASKTISKASENVKEQATTGAQIPVQPAAIPFKSVFDTQSSGDAKPPAA 240**

**Rpt 3 GEYGCSALPKEDASKTISKASENVKEQATTGAQIPVQPAATPFKSVFDTQSSGDAKPPAA 240**

****************************************** *********************

**Rpt 1 TPFKSVFDTQSSGDAKPPAATPFKSVFGTQSSGDAKPPAATPFKSVFGTQSSGDAKPPAA 300**

**Rpt 2 TPFKSVFGTQSSGDAKPPAATPFKSVFGTQSSGDAKPPAATPFKSVFGTQSSG------- 293**

**Rpt 3 TPFKSVFGTQSSGDAKPPAATPFKSVFGTQSSGDAKPPAATPFKSVFDTQSSGDAKPPAA 300**

*********.***************************************.*******

**Rpt 1 TPFKSVFDTQSSGDAKPPAATPFKSVFDTQSSGDAKPPAATPFKSVFGTQSSGDAKPPAA 360**

**Rpt 2 ------------------------------------------------------------**

**Rpt 3 TPFKSVFGTQSSGDAKPPAATPFKSVFDTQSSGDAKPPAATPFKSVFGTQSSGDAKPPAA 360**

**Rpt 1 TPFKSVFDTQSSGDAKPPAATPFKSVFGTQSSGDAKPPAATPFKSVFDTQSSGDAKPPAA 420**

**Rpt 2 -----------------------------------------------------DAKPPAA 300**

**Rpt 3 TPFKSVFGTQSSGDAKPPAATPFKSVFGTQSSGDAKPPAATPFKSVFDTQSSGDAKPPAA 420**

***********

**Rpt 1 TPFK---------------SDSINGFFIKQTRAGI------------------------- 440**

**Rpt 2 TPFK---------------SDSINGFFIKQTRAGI------------------------- 320**

**Rpt 3 TPFKSVFDTQSSGDAKPPAATPFKSVFGTQSSGDAKPPAATPFKSVFGTQSSGDAKPPAA 480**

****** : .::..* .*: ..**

**Rpt 1 ------------------------------------------------------------**

**Rpt 2 ------------------------------------------------------------**

**Rpt 3 TPFKSVFGTQSSGDAKPPAATPFKSVFGTQSSGDAKPPAATPFKSVFGTQSSGDANCRGG 540**

**Rpt 1 ------------------------------------------------------------**

**Rpt 2 ------------------------------------------------------------**

**Rpt 3 VVSICLDGESSVTNSASSSCRSTVQANILHDSVGGGVVCVADDLSGYQSNSLDEALNVTG 600**

**Rpt 1 ------------------------------------------------------------**

**Rpt 2 ------------------------------------------------------------**

**Rpt 3 LSPLFSAFERMMNDAKEKVLSALKEELNQMVSCVGGRDCCKCGRAIQSGREVKENYRGRN 660**

**Rpt 1 -------------------**

**Rpt 2 -------------------**

**Rpt 3 KSGDKIMWAHLENAFTKPY 679**

**(C) Comparing nucleotide sequences from the perfect repeat regions of TbNup149.**

**Rpt1 ATGTCTGAAATAGCTCACTCTGCGGTGGGTGACGTGCCGATTGACTCTTTTAAAGCAGAC 60**

**Rpt2 ATGTCTGAAATAGCTCACTCTGCGGTGGGTGACGTGCCGATTGACTCTTTTAAAGCAGAC 60**

**Rpt3 ATGTCTGAAATAGCTCACTCTGCGGTGGGTGACGTGCCGATTGACTCTTTTAAAGCAGAC 60**

****************************************************************

**Rpt1 AGTCACAATGGTGGAAAAGATGCATGGGTGTGCACCCGATGTGGTAAGGTGAAGGATCTT 120**

**Rpt2 AGTCACAATGGTGGAAAAGATGCATGGGTGTGCACCCGATGTGGTAAGGTGAAGGATCTT 120**

**Rpt3 AGTCACAATGGTGGAAAAGATGCATGGGTGTGCACCCGATGTGGTAAGGTGAAGGATCTT 120**

****************************************************************

**Rpt1 CGGGGTGATCATCTTCGAGGCAAGATGGAGGTCCGTTCTGATTGTTGGCCCTGTGGAAAG 180**

**Rpt2 CGGGGTGATCATCTTCGAGGCAAGATGGAGGTCCGTTCTGATTGTTGGCCCTGTGGAAAG 180**

**Rpt3 CGGGGTGATCATCTTCGAGGCAAGATGGAGGTCCGTTCTGATTGTTGGCCCTGTGGAAAG 180**

****************************************************************

**Rpt1 AAGGCGACATTCAAGTTGCAAAGCAGGCTCTCGGATGACGGTAGTAATAACAACGTCCCT 240**

**Rpt2 AAGGCGACATTCAAGTTGCAAAGCAGGCTCTCGGATGACGGTAGTAATAACAACGTCCCT 240**

**Rpt3 AAGGCGACATTCAAGTTGCAAAGCAGGCTCTCGGATGACGGTAGTAATAACAACGTCCCT 240**

****************************************************************

**Rpt1 GCAGCTAGCACTGTCGATGATACGCATCAACCAAAAGGCGTCGGGAACGCAACAATAGTT 300**

**Rpt2 GCAGCTAGCACTGTCGATGATACGCATCAACCAAAAGGCGTCGGGAACGCAACAATAGTT 300**

**Rpt3 GCAGCTAGCACTGTCGATGATACGCATCAACCAAAAGGCGTCGGGAACGCAACAATAGTT 300**

****************************************************************

**Rpt1 GGAGGATTTCGTGCTCCTGTCGTTCCTTCAACCGGTGACGAGCACCAGTTTCTTTTCCCT 360**

**Rpt2 GGAGGATTTCGTGCTCCTGTCGTTCCTTCAACCGGTGACGAGCACCAGTTTCTTTTCCCT 360**

**Rpt3 GGAGGATTTCGTGCTCCTGTCGTTCCTTCAACCGGTGACGAGCACCAGTTTCTTTTCCCT 360**

****************************************************************

**Rpt1 GCTCCCGTACTCCAGTGCTCAACCCCAAGCTTATCAGCAGACTGCTCCTCTGTATGGAAA 420**

**Rpt2 GCTCCCGTACTCCAGTGCTCAACCCCAAGCTTATCAGCAGACTGCTCCTCTGTATGGAAA 420**

**Rpt3 GCTCCCGTACTCCAGTGCTCAACCCCAAGCTTATCAGCAGACTGCTCCTCTGTATGGAAA 420**

****************************************************************

**Rpt1 TGCTCTACCTGTGGAAAGGTGAAGCCACTTTCTGGTGATCATTTGCGGGGGAAAACAGAG 480**

**Rpt2 TGCTCTACCTGTGGAAAGGTGAAGCCACTTTCTGGTGATCATTTGCGGGGGAAAACAGAG 480**

**Rpt3 TGCTCTATCTGTGGAAAGGTGAAGCCACTTTCTGGTGATCATTTGCGGGGGAAAACAGAG 480**

********* ******************************************************

**Rpt1 GTGCGAAGCGATTGCTGGCCTTGTGGCTGCAAGAGAACGTTTGTGTTGAGTCCTGTTGAC 540**

**Rpt2 GTGCGAAGCGATTGCTGGCCTTGTGGCTGCAAGAGAACGTTTGTGTTGAGTCCTGTTGAC 540**

**Rpt3 GTGCGAAGCGATTGCTGGCCTTGTGGCTGCAAGAGAACGTTTGTGTTGAGTCCTGTTGAC 540**

****************************************************************

**Rpt1 GGTGAATACGGATGCTCCGCGTTGCCGAAGGAGGATGCATCGAAGACTATTTCAAAGGCC 600**

**Rpt2 GGTGAATACGGATGCTCCGCGTTGCCGAAGGAGGATGCATCGAAGACTATTTCAAAGGCC 600**

**Rpt3 GGTGAATACGGATGCTCCGCGTTGCCGAAGGAGGATGCATCGAAGACTATTTCAAAGGCC 600**

****************************************************************

**Rpt1 AGCGAGAACGTTAAGGAGCAAGCTACCACTGGCGCACAAATACCAGTACAACCCGCAGCA 660**

**Rpt2 AGCGAGAACGTTAAGGAGCAAGCTACCACTGGCGCACAAATACCAGTACAACCCGCAGCA 660**

**Rpt3 AGCGAGAACGTTAAGGAGCAAGCTACCACTGGCGCACAAATACCAGTACAACCCGCAGCA 660**

****************************************************************
