# Supplementary material for: Interactome Mapping Reveals the Evolutionary History of the Nuclear Pore Complex
Source: PLoS Biol. 2016 Feb 18;14(2):e1002365. doi: 10.1371/journal.pbio.1002365 (PMC4758718; doi:10.1371/journal.pbio.1002365)
Supplement: S4 Table — There appear to be three major FG-Nup flavors in trypanosomes. Interestingly, the inner and outer ring FG-Nups all share the same GFG flavor. Likewise, the two Nup76 FG-Nups have SVFG or PAFG flavors (predominantly PAFG for Nup140). The multi complex FG-Nups also have a shared FSFG flavor. (DOCX) [file pbio.1002365.s013.docx]

| Gene ID | FG-Nup | No. of FGs | Major FG flavor | NPC subcomplex |
| --- | --- | --- | --- | --- |
| Tb927.11.15560 | TbNup53a | 13 | 9xGFG | Inner ring |
| Tb927.3.3540 | TbNup53b | 11 | 8xGFG | Inner ring |
| Tb927.4.5200 | TbNup62 | 30 | 29xGFG | Inner ring |
| Tb927.11.980 | TbNup158 | 58 | 51xGFG | Outer ring |
| Tb927.11.11090 | TbNup140 | 101 | 11xSVFG  81xPAFG | Nup76 complex |
| Tb927.11.11080 | TbNup149 | 18 | 18xSVFG | Nup76 complex |
| Tb927.4.4310 | TbNup64^#^ | 13 | 9xFG, 4xFSFG | Multiple complexes |
| Tb927.8.8050 | TbNup75^#^ | 10 | 6xFG, 3xFSFG | Multiple complexes |
| Tb927.3.3180 | TbNup98 | 21 | 20xFSFG | Multiple complexes |
